# Laser‐Enabled Fabrication of Flexible Printed Electronics with Integrated Functional Devices

**DOI:** 10.1002/advs.202415272

**Published:** 2025-03-04

**Authors:** Wedyan Babatain, Christine Park, Hiroshi Ishii, Neil Gershenfeld

**Affiliations:** ^1^ Media Lab Massachusetts Institute of Technology Cambridge MA 02139 USA; ^2^ Department of Electrical Engineering and Computer Science Massachusetts Institute of Technology Cambridge MA 02139 USA; ^3^ Center for Bits and Atoms Massachusetts Institute of Technology Cambridge MA 02139 USA

**Keywords:** additive manufacturing, digital fabrication, flexible electronics, laser writing, printed electronics

## Abstract

The demand for flexible and printed electronics in wearable and soft robotics applications has increased the need for scalable, additive manufacturing processes. However, traditional printed circuit board manufacturing involves complex, multistep processes, is limited to certain substrates, and faces challenges in integrating functional devices. Here, an additive, laser‐enabled process is introduced for fabricating flexible, double‐sided printed electronics leveraging laser‐induced graphene (LIG) as a seed layer for selective copper electrodeposition (E‐LIG). This technique enables precise conductive circuit patterning down to 50 µm and is reliable via formation in a single streamlined process. E‐LIG supports transfer to various substrates, allowing for large‐area electronics up to 100 cm^2^, broadening applications in large‐scale interfaces. Functional LIG device integration, including sensors and actuators, directly interfaced with control circuits on a single substrate is demonstrated. Applications such as real‐time graphical output and interactive interfacing showcase the method's versatility. E‐LIG exhibits repairability for on‐demand restoration of damaged circuits, enhancing durability and offering a scalable, cost‐effective solution for multifunctional printed electronics.

## Graphical abstract

The developed laser‐enabled selectively electroplated laser‐induced graphene fabrication process enables double‐sided, large‐area, flexible printed electronics with integrated functional devices. This additive method achieves precise circuit patterning, robust via interconnects, and is easy to transfer to various substrates. Demonstrating on‐demand repairability, it supports seamless integration with functional devices, offering a scalable solution for durable, multifunctional electronics in sensing and interactive applications.

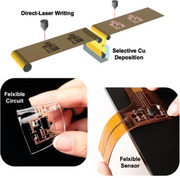

## 1 Introduction

Flexible and printed electronics are a key component in the next generation of microelectronic systems, particularly for wearable devices, soft robotics, and transparent electronics.^[^ ^1^, ^2^, ^3^, ^4^, ^5^ ^]^ These applications require mechanical compliance, miniaturization, and integration with conventional silicon‐based electronics. Traditional manufacturing methods of printed circuit boards (PCBs) and silicon‐based electronics rely on subtractive techniques such as photolithography and etching, resulting in material waste and multiple fabrication steps.^[^ ^6^, ^7^ ^]^ Additionally, creating double‐sided connections and vias is time‐consuming and inefficient. Moreover, conductive layers on some polymeric substrates often exhibit poor adhesion and are incompatible with high thermal processes. Additive manufacturing has emerged as an alternative to traditional fabrication methods, offering reduced material waste, design customizability, substrate versatility, and high‐volume manufacturing.^[^ ^8^ ^]^ Techniques like inkjet printing,^[^ ^9^, ^10^ ^]^ direct ink writing,^[^ ^11^, ^12^ ^]^ screen printing,^[^ ^13^, ^14^ ^]^ spray coating,^[^ ^15^, ^16^ ^]^ and 3D printing^[^ ^17^, ^18^ ^]^ have demonstrated potential for electronics fabrication and processing. However, these methods suffer from challenges including limited functional ink options, ink–substrate interaction, ink preparation, and suboptimal ink conductivity.^[^ ^9^, ^10^, ^19^ ^]^ Direct laser writing (DLW) is a promising solution due to its ability to pattern materials with high precision and efficiency,^[^ ^20^, ^21^, ^22^, ^23^ ^]^ and to combine multiple fabrication steps into one process including material conversion and synthesis. Laser‐induced graphene (LIG), synthesized by laser irradiation of carbon‐rich materials, exhibits good electrical conductivity, flexibility, scalability, and versatility, making it suitable for flexible and printed electronics.^[^ ^24^, ^25^, ^26^, ^27^ ^]^ LIG has been applied in various functional devices, including physical and electrochemical sensors,^[^ ^28^, ^29^, ^30^ ^]^ actuators,^[^ ^31^, ^32^, ^33^ ^]^ and energy storage and harvesting devices.^[^ ^34^, ^35^, ^36^ ^]^ However, its applications are limited by its relatively high resistivity compared to traditional metals like copper. Recent techniques like flash healing^[^ ^37^ ^]^ have improved LIG's conductivity, but still fall short of achieving the performance required for highly conductive electronics. In this work, we introduce selectively electroplated laser‐induced graphene (E‐LIG) as a scalable, laser‐enabled process for fabricating flexible, double‐sided printed electronics with highly conductive traces and integrated functional devices. By combining the additive nature of DLW with electroplating, our method overcomes LIG's conductivity limitations, transforming it into a viable material for highly conductive electronics. E‐LIG broadens the scope of LIG applications and simplifies the fabrication of flexible, printed electronics with integrated functional devices. E‐LIG structures can also be transferred to flexible and transparent substrates, making the method suitable for wearable electronics and human–machine interfaces. While LIG has been used as a seed layer for electroplating contacts,^[^ ^38^, ^39^ ^]^ an optimized streamlined process flow for fabricating full circuit boards using LIG remains unexplored. Moreover, our process leverages DLW to directly create double‐sided PCBs using conductive vias in a single step, reducing complexity and material waste associated with traditional methods. As a complementary advantage, E‐LIG enables on‐demand circuit repair by leveraging DLW to relase the carbon‐rich substrate, allowing damaged circuits to be restored on demand, restoring functionality. Furthermore, the scalability of E‐LIG supports large‐area electronics, enabling flexible circuits to be fabricated across substantial surface areas with precision and customizability. A key advantage of our process is its ability to integrate functional LIG devices with their circuits on a single substrate, improving integration, simplifying assembly, and addressing a major challenge in the field of LIG‐based devices by creating homogeneous electrical contact between functional devices and their circuits. These features position E‐LIG as a versatile, additive, laser‐enabled solution for advancing multifunctional printed electronics across applications, from wearable technology to soft robotics.

## 2 Results and Discussion

### 2.1 Fabrication Process of E‐LIG Printed Electronics

**Figure 1:**
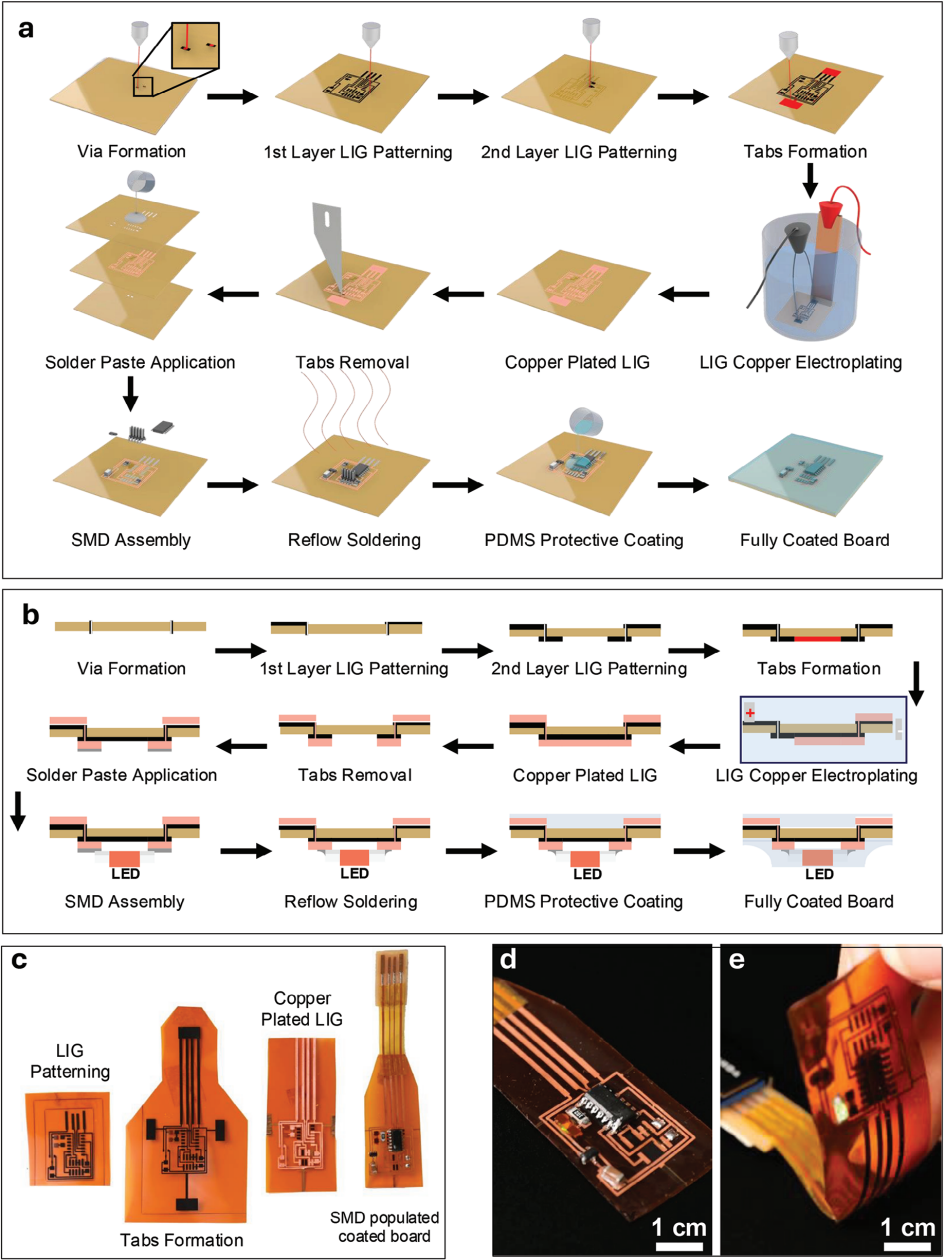
Process flow of the E‐LIG fabrication method for printed electronics. a) Schematic illustration of the full streamlined fabrication process including LIG patterning, from two sides followed by via and tab formation. After copper plating, the tabs are removed, solder paste is applied, and surface mount devices (SMDs) are assembled. b) Cross‐sectional diagram illustrating the step‐by‐step development of the PCBs. c) Photographs showing the progression on the original polyamide substrate including the intermediate and final stages of the PCB fabrication process. d) Photograph of the fully assembled and functional double‐sided flexible PCB with e) a LED on the bottom layer.

The streamlined E‐LIG fabrication process is illustrated in **Figure** **1**. As shown in Figure 1a, the process begins with the irradiation of a polyimide (PI) substrate using a CO_2_ laser, which directly forms a conductive graphitic layer. First, vias are formed by cutting holes through the substrate at predefined locations, allowing for the formation of a LIG ring around the edges of the ablated holes. This LIG ring establishes conductive pathways between the two sides of the PCB. The first circuit layer is then patterned with LIG, after which the substrate is flipped and aligned to pattern the second LIG layer on the opposite side. To prepare for electroplating, LIG tabs (highlighted in red) (Figure S1, Supporting Information) are formed to establish common grounding connections to all pads and traces, facilitating uniform selective electrodeposition. The substrate is immersed in an electroplating solution and the optimal plating parameters ensure even deposition of copper onto the LIG patterns, resulting in highly conductive E‐LIG traces. Once copper fully plates the pattern, the tabs are removed either manually with a scalpel or by laser ablation. Next, low‐temperature solder paste is applied through a polyimide mask, followed by surface mount device (SMD) assembly and reflow soldering. The final step involves applying a silicone coating, polydimethylsiloxane (PDMS) to enhance mechanical durability. The PDMS coating acts as a protective layer, preventing delamination during deformation (Video S12, Supporting Information). Figure 1b shows a cross‐sectional view of the process, illustrating the layer‐by‐layer buildup of the board. Figure 1c captures photographs of the progression from LIG patterning alone to the final copper‐plated assembly. The fully assembled, functional double‐sided printed board is depicted in Figure 1d, with a microcontroller on one side and a blinking Light ‐Emitting Diode (LED) on the other (Figure 1e). The overall process is highly efficient, allowing for the rapid production of flexible, double‐sided PCBs with integrated functional devices (Video S1, Supporting Information).

### 2.2 Material and Physical Characterization

**Figure 2:**
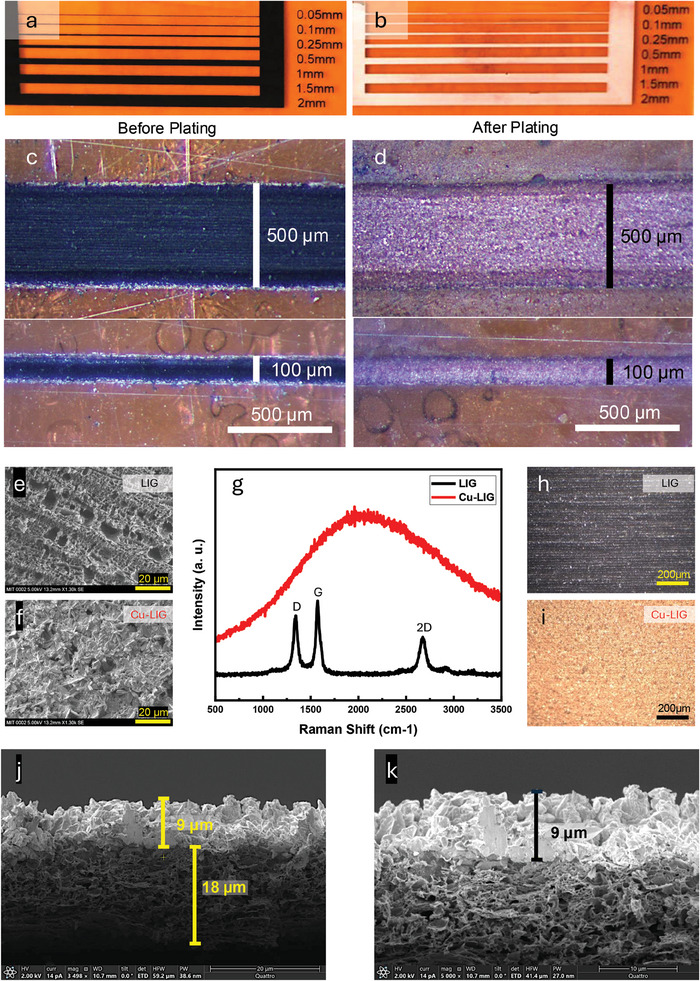
Material and physical characterization of E‐LIG traces. a) Optical images of LIG traces on polyimide showing various line widths before and b) after copper plating ranging from 50 µm to 2 mm. c) Zoomed‐in microscopic image of 500 and 100 µm thick LIG traces before and d) after copper plating demonstrating the uniformity of copper deposition across the same trace width. e) Scanning electron microscope (SEM) image of the surface of LIG trace showing its porous graphitic nature. f) SEM image of the copper‐plated LIG showing morphological changes after Cu deposition. g) Raman spectroscopy of LIG and Cu–LIG. h) Microscopic images showing a top view of the surface of LIG and i) Cu–LIG. j,k) Cross‐sectional SEM images of copper‐plated LIG layer, showing a thickness of ≈9 µm for Cu and 18 µm for LIG.

To ensure the reliability of the E‐LIG PCBs, a series of material and electrical characterizations was performed, including optical microscopy, scanning electron microscope (SEM), Raman spectroscopy, and resistance measurements. Our method demonstrates the ability to fabricate E‐LIG as small as 50 µm in width while maintaining structural and electrical integrity, critical for high‐resolution flexible electronics and matching commercial standard PCB manufacturing capabilities.^[^ ^40^, ^41^ ^]^ **Figure** **2**a,b shows the LIG traces before and after copper plating, respectively. The consistent width and well‐defined edges across the range of trace widths (50 µm to 2 mm) highlight the precision of the DLW patterning and subsequent copper plating processes. A closer view of the trace morphology is shown in the microscopic images of Figure 2c,d. The copper deposition is uniform across the trace surface, indicating uniform LIG seeding and resistance resulting in effective subsequent electroplating. SEM images in Figure 2e,f provide further insights into surface material morphology before and after copper plating. The LIG surface (Figure 2e) exhibits a characteristic porous nature typical of graphitic structures.^[^ ^24^ ^]^ After copper plating (Figure 2f), the surface morphology undergoes a significant transformation, with well‐defined crystalline structures, suggesting sparse nucleation of copper followed by rapid crystal growth, likely driven by high current densities during plating.^[^ ^42^ ^]^ Raman spectroscopy (Figure 2g) confirms the successful formation of LIG and its subsequent copper plating. The LIG spectrum displays characteristic D, G, and 2D bands, with the G band suggesting the formation of stacked graphene layers. Postplating, there is a marked reduction in LIG peak intensity, consistent with complete copper coverage.^[^ ^24^ ^]^ This is further corroborated by top‐view microscopy in Figure 2h,i, which corresponds to a top‐view of the samples that were used to obtain the Raman spectra. The cross‐sectional SEM images (Figure 2j,k) show a uniform LIG layer with a thickness of ≈18 µm and a copper layer with a thickness of ≈9 µm. The conductivity of these layers was measured to be 1.789 × 10^6^ S m^−1^ for LIG and 4.16 × 10^6^ S m^−1^ for Cu–LIG (Note S3, Supporting Information)

### 2.3 Electrical Characterization

**Figure 3:**
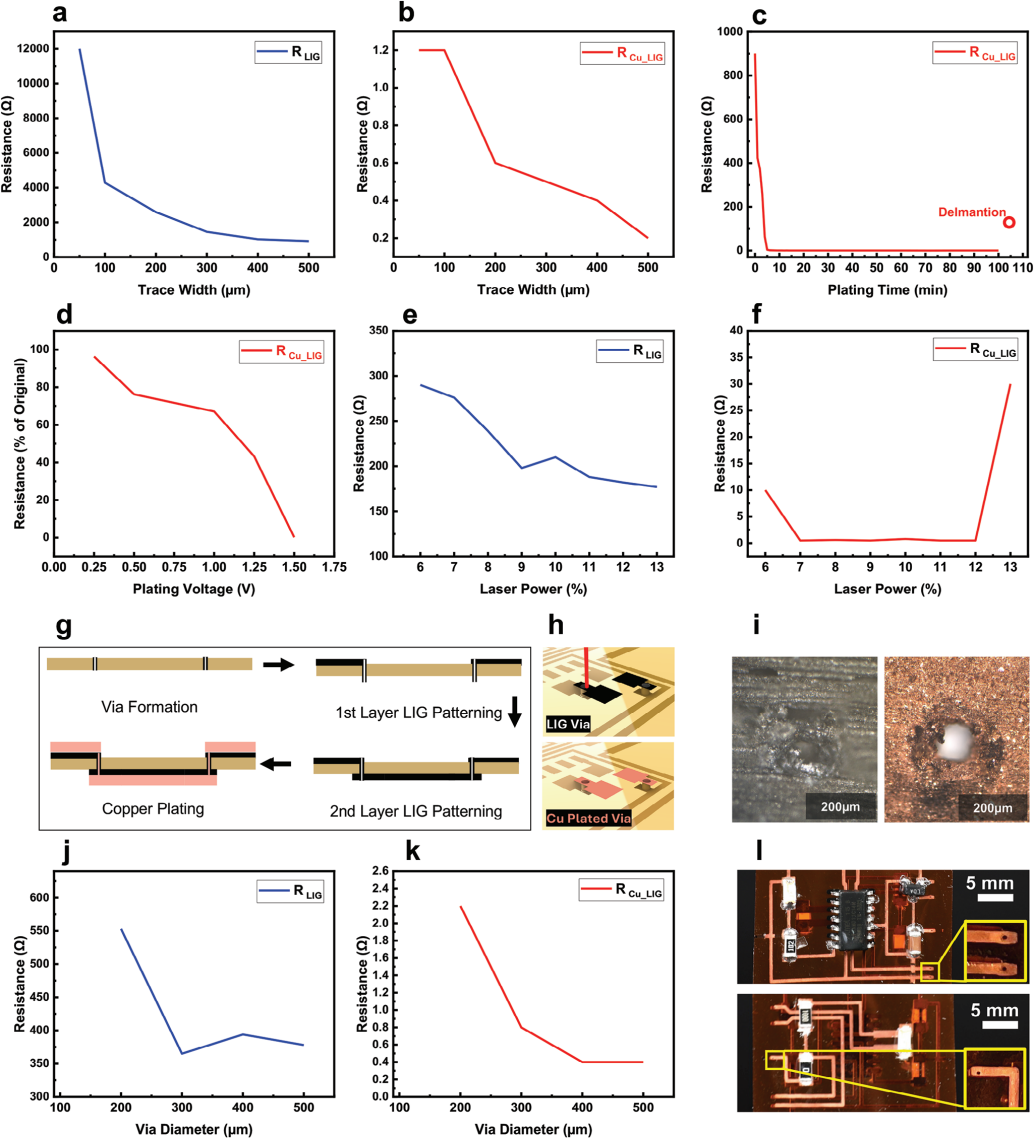
Electrical characterization of single and double‐sided E‐LIG traces as a function of process parameters. a) Resistance of LIG and b) Cu–LIG as a function of trace width. c) Resistance of Cu–LIG over time during the plating process. d) Resistance of Cu–LIG as a percentage of the original LIG resistance as a function of plating voltage, illustrating the optimal voltage range. e) Resistance of LIG and f) Cu–LIG as a function of laser power. g) Schematic of the process flow for creating double‐sided PCBs, including the LIG patterning, via formation, and Cu deposition to establish interconnections between layers. h) 3D illustration of the process of formation of LIG vias and the subsequent copper plating to create conductive paths between the two sides of the PCB. i) Microscopic images of the LIG vias before and after copper plating, showing the formation of reliable Cu electrical connections in double‐sided PCBs. j) Resistance of LIG and k) Cu–LIG as a function of via diameter. l) Photographs showing a fully Cu‐plated double‐sided trace enabled by the formation of LIG vias, with insets showing zoomed‐in views of the vias on both sides.

To optimize the electroplating conditions and process parameters, we conducted comprehensive characterization to evaluate the effect of trace width, plating time, and laser settings on the electrical properties of the resulting E‐LIG traces. **Figure** **3**a shows resistance measurements for 20 mm long LIG traces with widths ranging from 50 to 500 µm. As expected, wider traces exhibit lower resistance due to the increased cross‐sectional area for current flow. LIG, being moderately conductive, exhibits relatively high resistance for narrow traces (1–12 kΩ), rendering them unsuitable for direct circuit use. After electroplating, resistance decreases by up to four orders of magnitude (Figure 3b), demonstrating the effectiveness of copper electroplating in enhancing conductivity (Figure S2, Supporting Information). To determine the optimal plating time, a 2 mm × 80 mm LIG trace was plated for durations ranging from 1 to 110 min at a constant voltage of 1 V. The resistance measurements (Figure 3c) reveal that most resistance reduction occurs within the first 10 min, with diminishing returns thereafter. Beyond 30 min, excessive copper deposition leads to a spongy texture that is prone to damage, with delamination occurring at 110 min denoted with a hollow circle in Figure 3c. The influence of electroplating voltage was also evaluated. At a fixed plating time of 5 min, 2 mm × 80 mm LIG traces were plated at voltages from 0.25 to 1.5 V. As shown in Figure 3d, higher voltages yield greater resistance reduction, with 1.25 and 1.5 V being most effective. However, higher voltages can lead to uneven deposition, making lower voltages (0.25–0.5 V) more desirable for uniform coating. Additional optical images showing the effect of lower voltages and plating uniformity are shown in Figure S17 (Supporting Information). Finally, the effect of CO_2_ laser settings on trace quality and subsequent plating was assessed. At a fixed laser speed of 10%, traces were patterned using powers from 6% to 13% of the laser's maximum 30 W output. Figure 3e shows resistance values of the initial traces and Figure 3f shows resistance after plating for 5 min at 1 V. Optimal laser power exists between 7% and 12%, producing traces with good conductivity and uniform deposition. Below 7%, higher resistance requires longer plating, while above 12%, traces become flaky and unstable hindering uniform deposition. The results confirm the importance of fine‐tuning both laser parameters and electroplating conditions to achieve uniform, highly conductive E‐LIG traces suitable for various PCB applications.

### 2.4 Double‐Sided PCBs Enabled by E‐LIG Vias

We introduce a novel approach for establishing electrical connections between PCB layers using LIG vias. Traditional via fabrication involves multiple steps like hole drilling, electroless plating, and electroplating, which are time‐consuming and material‐intensive. Our method simplifies this process by leveraging the laser ablation of polyimide to form a conductive LIG ring around the ablated hole, creating a via that connects the top and bottom PCB layers. Previous work demonstrated direct‐write LIG for bottom contact but with high resistance.^[^ ^43^ ^]^ By contrast, our process plates the LIG via with copper, forming a reliable highly conductive connection. Figure 3g illustrates the process where initially a through‐hole is created at the desired location using laser cutting. The top LIG pad is then patterned on one side of the substrate, the second LIG pad is patterned on the opposite side, forming a via with a conductive LIG rim that connects the top and bottom pads. Microscopic images in Figure 3i show the formed via before and after copper plating, with the copper fully covering the porous LIG surface to form a continuous conductive path bridging the two sides. This automatic one‐step transition from a resistive LIG via to a conductive E‐LIG via is critical for double‐sided PCB functionality. Initially, the LIG via exhibits high resistance, insufficient for PCB applications. However, after copper plating, the LIG rim serves as a seed layer, and the copper seamlessly covers both sides, transforming the resistive LIG into a highly conductive via. A 3D representation of the via formation process is shown in Figure 3h. We experimented with via diameters from 50 to 500 µm. Vias smaller than 200 µm did not reliably plate, likely due to insufficient surface area for copper deposition. Vias larger than 200 µm demonstrated optimal performance, with lower resistance, as shown in Figure 3j,k while smaller vias exhibit higher resistance due to the limited cross‐sectional area available for current flow. Figure 3l shows a fully assembled double‐sided PCB with inset images providing a closer view of the conductive vias on both sides. This E‐LIG‐enabled via process (Video S2, Supporting Information) reduces fabrication steps and integrates via creation within the DLW process, improving efficiency and opening new possibilities for high‐performance printed circuits.

### 2.5 Transfer of Printed Circuits onto Transparent and Large‐Area Substrates

**Figure 4:**
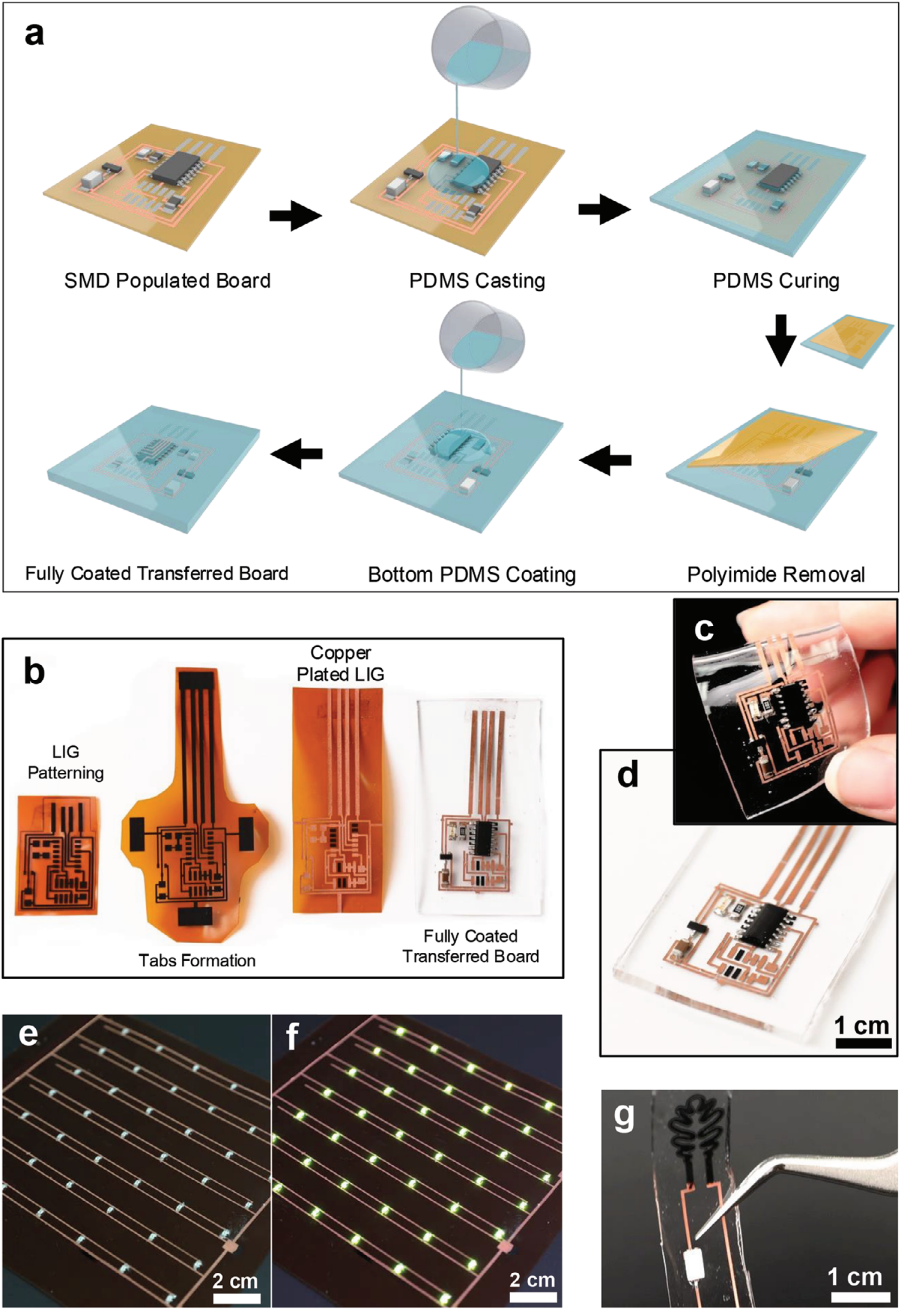
Fabrication and transfer of printed circuits onto transparent and large‐area substrates. a) Process flow illustration of the step‐by‐step process for transferring a fully plated and assembled PCB onto a PDMS substrate. b) Sequence of photographs showing the intermediate and final stages of the PCB transfer process. c,d) Photographs of the transferred PCB on PDMS showing the uniformity of the transfer process and the flexibility of the transferred PCB on PDMS. e,f) A large‐area (10 cm × 10 cm) LED matrix fabricated on using the E‐LIG method, demonstrating the scalability of the process. g) Transferred PCB on PDMS integrated with a LIG resistive sensor controlling the brightness of a LED, showing seamless functional integration.

A key advantage of utilizing LIG as a seed layer in our fabrication method is its ability to transfer onto various substrates, including elastomers, forming multifunctional composites.^[^ ^44^, ^45^, ^46^ ^]^ This feature not only enhances the physical robustness of LIG‐based circuits but also expands their versatility across applications. Our process allows E‐LIG PCBs to be transferred onto flexible, stretchable, or transparent substrates like PDMS, which are ideal for flexible electronics and wearables. **Figure** **4**a illustrates the transfer process, where the fully populated board is cast with a PDMS layer. During the curing phase, PDMS infiltrates the porous LIG structure, creating a strong bond and forming a composite material. After curing, the polyimide substrate is peeled off, leaving the E‐LIG traces and embedded components intact within the PDMS (Figure S3, Supporting Information). A final thin PDMS layer is then cast onto the exposed side, encapsulating the entire board and rendering it fully coated. Photographs in Figure 4b show the intermediate steps from LIG patterning and Cu plating to the final transferred board embedded in PDMS. Figure 4c,d demonstrates the flexibility and transparency of the substrate, highlighting the versatile potential of the method for various nontraditional substrates. Figure 4e,f demonstrates the scalability of the method, showcasing a large‐area (10 cm × 10 cm) LED matrix consisting of 42 individual LEDs. This demonstrates the feasibility of producing large‐scale electronics using this process.^[^ ^47^, ^48^ ^]^ Additionally, Figure 4g shows a transferred circuit, integrating a functional LIG resistive sensor that controls LED brightness, demonstrating seamless circuit integration with functional LIG elements.

### 2.6 Application of E‐LIG Integrated Printed Electronics

#### 2.6.1 Sensors

**Figure 5:**
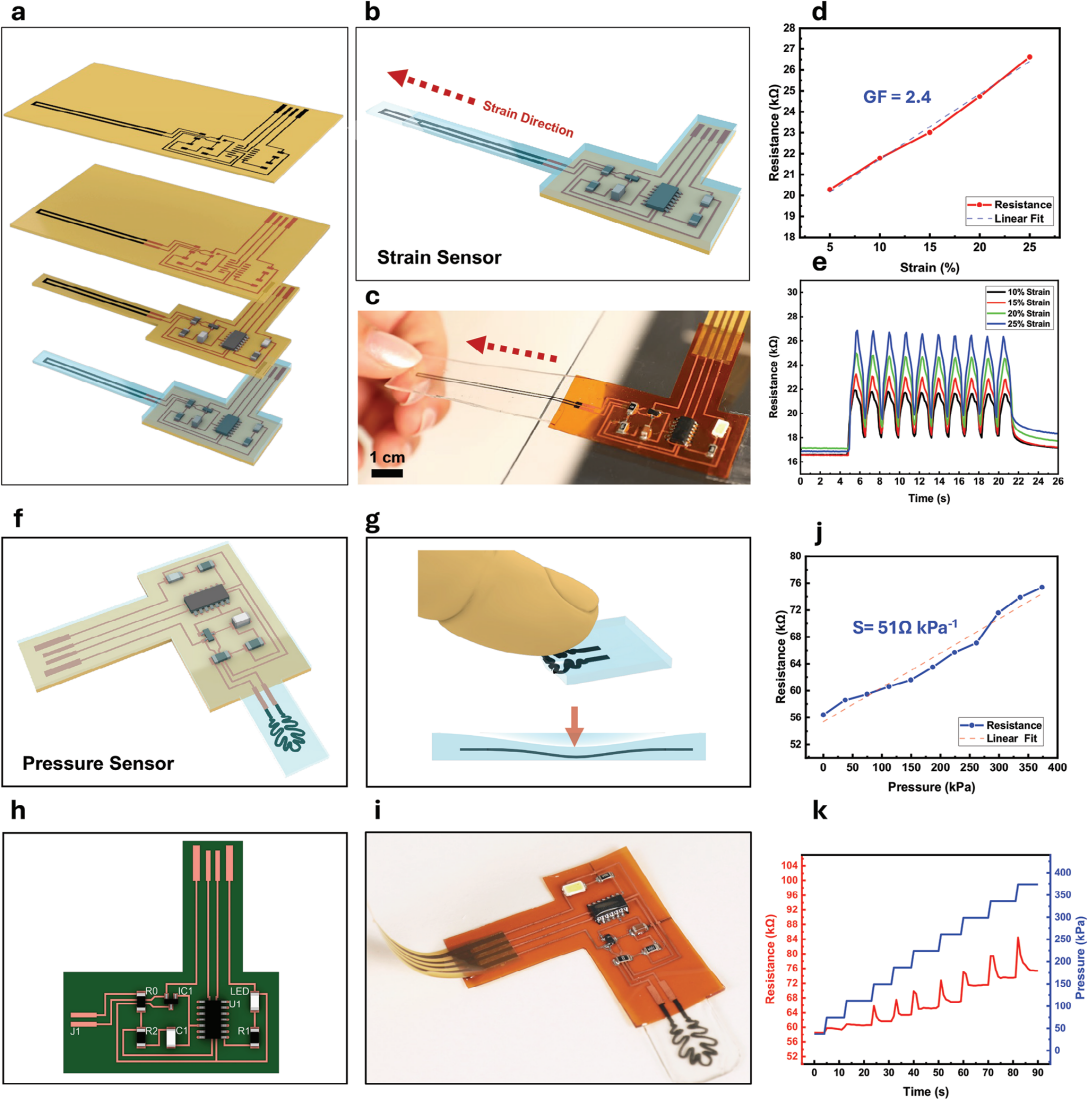
E‐LIG integrated electronics for sensor applications. a) Schematic illustration of the layer‐by‐layer fabrication process of E‐LIG board with integrated functional LIG strain sensor. b) Schematic and c) photograph of the strain sensing integrated system including the sensing element and its readout circuit in one substrate. d) Correlation of the resistance of the sensing element with the overall strain applied from 5% to 25%. e) Resistance of the sensor during a cyclic test of 10 repeated strain cycles at various strain values. f) Schematic representing the integrated LIG pressure sensor, including the pressure‐sensitive element and readout circuit. g) Schematic showing how external pressure is applied to the sensor, causing a change in resistance due to its piezoresistive behavior. h) Layout of the readout circuit designed for sensor interface. i) Physical image of the integrated LIG‐based pressure sensor system. j) The resistance–pressure characteristic curve demonstrates a linear increase in resistance as pressure increases. k) Dynamic resistance response of the sensor under multiple increasing pressure cycles.

Despite the recent proliferation of LIG‐based devices such as sensors, actuators, and energy harvesters,^[^ ^26^, ^28^, ^29^, ^31^, ^32^, ^33^, ^34^, ^35^, ^49^ ^]^ reliably connecting these functional devices with circuits remains challenging. High contact resistance and nonhomogeneous integration often hinder performance, while introducing rigid pastes like silver epoxy can impose mechanical vulnerability and limit flexibility (Figure S4, Supporting Information).^[^ ^50^ ^]^ Our proposed method addresses this long‐standing issue by offering seamless integration of LIG functional devices with E‐LIG PCBs in a single substrate and fabrication step. This is enabled by selectively protecting the LIG functional part from plating either by controlled immersion in the solution or by physical coating (Figure S5, Supporting Information). This selectivity enables turnability of trace conductivity and even replaces SMD components such as a LED current limiting resistor with a printed resistor (Figure S6, Supporting Information). **Figure** **5**a schematically illustrates a flexible LIG strain sensor integrated into an E‐LIG PCB, combining a LIG/PDMS resistive element with a voltage divider readout circuit based on SAMD11 microcontroller (Figure S7 and Table S1, Supporting Information). The strain sensor works by increasing resistance as strain is applied, separating the LIG particles further apart due to the strain induced by the deformation (Figure 5b). A photo of the integrated flexible strain sensing system is shown in Figure 5c. The strain sensor exhibits a gauge factor (GF) of 2.4, as shown in the linear resistance–strain correlation in Figure 5d, which compares favorably with conventional strain gauges.^[^ ^51^ ^]^ Figure 5e demonstrates the sensor's stable performance across multiple strain cycles of different percentages. The performance of the strain sensor, including LED intensity control and graphical data visualization using the E‐LIG board, is shown in Videos S3–S5 (Supporting Information). Another demonstration of our developed E‐LIG PCB fabrication method was used to develop a LIG‐based pressure sensing system (Figure 5f). The sensor leverages the piezoresistive effect of LIG, where applied pressure compresses the LIG's porous structure, changing its conductive pathways and leading to a measurable resistance change^[^ ^52^ ^]^ (Figure 5g). The LIG was fabricated with a porous structure to enhance pressure sensitivity and fully transferred to a PDMS substrate, while the readout circuit remained on the original polyimide substrate. The sensor exhibits a pressure sensitivity of 51 Ω kPa^−1^ (Figure 5j), making it highly suitable for applications in tactile sensing or wearable pressure monitoring. Circuit design and physical photographs of the fully integrated pressure sensing system are shown in Figure 5h,i, respectively. The time‐series response in Figure 5k further illustrates the sensor's ability to detect dynamic pressure changes with transient spikes observed during initial pressure application which may be attributed to either the initial compression and relaxation of the porous structure or the viscoelastic behavior of PDMS. Real‐time data logging and visualization of the pressure sensor performance using the integrated E‐LIG system can be found in Videos S6–S8 (Supporting Information).

#### 2.6.2 Actuators

**Figure 6:**
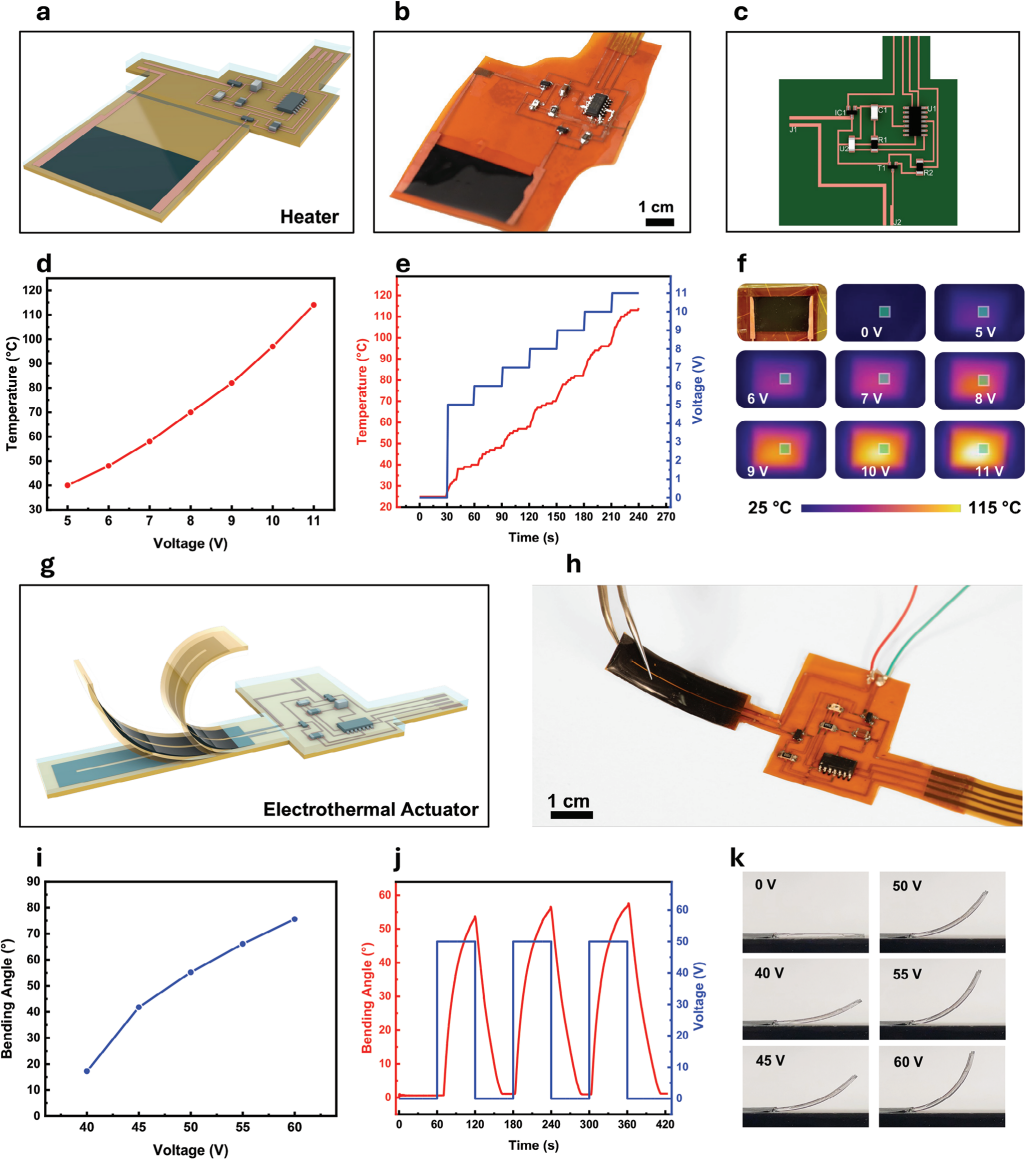
E‐LIG integrated electronics for actuator applications. a) Schematic illustration of the E‐LIG fabricated board with an integrated thin‐film LIG heater. b) Photograph of the LIG‐based heater integrated with its control circuit on the same substrate. c) Layout of the circuit design for the heater interface and control. d) Temperature versus voltage characteristics of the heater show an increase in temperature with increasing voltage. e) Dynamic response of the heater during incremental voltage steps. f) Infrared thermal images showing the temperature distribution across the LIG heater at different voltages. g) Schematic of the LIG‐based electrothermal actuator integrated with an E‐LIG board. h) Photograph of the LIG‐based electrothermal actuator integrated system. i) Bending angle of the actuator as a function of the applied voltage. j) Dynamic actuation response of the LIG‐based actuator under cyclic voltage application driven by a 50 V square wave. k) Sequential images showing the physical bending of the electrothermal actuator at different applied voltages (0–60 V).

LIG has proven versatile beyond sensing, extending to applications such as heaters, electrothermal actuators, and more.^[^ ^53^ ^]^ Joule heating, where resistive materials generate heat through the self‐heating effect, enables thin, flexible, and robust heating elements.^[^ ^54^, ^55^ ^]^ These are essential in microfluidics, gas sensing, and other applications.^[^ ^56^ ^]^ Traditional heaters, often based on silicon micromachining technologies, are effective but costly. With the advent of flexible electronics, alternative methods like LIG, which can be directly patterned onto polyimide substrates, offer scalable, cost‐effective solutions. An integrated heater and E‐LIG printed electronic system are shown in **Figure** **6**a, with a prototype depicted in Figure 6b. The PCB design of the control board is shown in Figure 6c. More details of the PCB design and components are summarized in Figure S8 and Table S2 (Supporting Information). The relationship between the applied voltage and the generated temperature of the heater is shown in Figure 6d, demonstrating a linear correlation. Figure 6e shows the transient response of the heater as the applied voltage is gradually increased every 30 s, highlighting the heater's responsiveness. Finally, infrared thermal images in Figure 6f show uniform temperature distribution across the heater under various voltages, demonstrating consistent heating performance. Programmed activation of the heater mapped to a phototransistor threshold in a close‐loop manner using the E‐LIG PCB is demonstrated in Video S9 (Supporting Information). Building on the heating functionality, LIG is also utilized in electrothermal actuators, enabling shape transformations through Joule heating.^[^ ^31^, ^33^ ^]^ This effect is driven by the thermal expansion mismatch between polyimide and PDMS layers in a multilayered stack, as shown in the schematic in Figure 6g. This difference facilitates controlled bending and folding in these actuators. The photo in Figure 6h shows the flexible electrothermal actuator integrated with its control E‐LIG PCB, showcasing untethered seamless integration. The actuator can bend upon the application of voltage, showcasing its flexibility and the seamless integration of LIG‐based circuits on a flexible substrate. The relationship between the applied voltage and the bending angle of the actuator is shown in Figure 6i, with higher voltages leading to greater bending angles. The dynamic response of the actuator during cyclic voltage application is depicted in Figure 6j, where consistent bending is observed over multiple cycles, indicating the actuator's repeatability. Finally, Figure 6k shows sequential images capturing the actuator's physical bending at voltages ranging from 0 to 60 V. As the voltage increases, the actuator progressively bends, confirming the effectiveness of the integrated PCB in controlling the actuator's motion. The programmed activation and operation of the electrothermal actuator using the E‐LIG circuit are shown in Video S10 (Supporting Information). These results demonstrate the versatility of our E‐LIG PCB fabrication process, enabling seamless integration of various LIG sensors and actuators with their interface/control circuits, showcasing its potential for a wide range of applications. The E‐LIG process also includes a repairability feature, allowing damaged circuits to be restored on demand by relasing the substrate (Figure S10 and Video S11, Supporting Information). This method quickly repairs broken connections, restoring functionality after issues like overheating, and extending the lifespan and durability of flexible electronics in challenging environments.

## 3 Conclusion

In this work, we introduced an additive laser‐enabled process for fabricating flexible, double‐sided printed electronics with integrated functional devices using LIG as a seed layer for selective copper electroplating. This method offers significant advancements over traditional PCB manufacturing techniques by providing a scalable, streamlined, and cost‐effective alternative. By eliminating complex multistep processes and reducing material waste, our approach enables the direct fabrication of intricate patterns with high precision onto flexible substrates. A key feature of this process is the creation of double‐sided PCBs through laser‐induced conductive vias, achieved in a single step. LIG is formed around the via edges and electroplated with copper, establishing seamless electrical connections between the two sides of the board. This eliminates the need for conventional, multistep via fabrication, reducing complexity and enhancing scalability. Furthermore, the versatility of E‐LIG extends to its transferability onto various substrates, broadening its application scope to wearable electronics, soft robotics, and transparent devices. The method allows for the fabrication of large‐area electronics without sacrificing precision or performance. A major advantage of our process is its ability to integrate functional LIG devices, such as sensors, heaters, and actuators, with their associated circuits in a single fabrication step. This resolves a long‐standing issue in LIG‐based electronics of high‐contact resistance and mechanical vulnerability in traditional methods, eliminating the need for rigid pastes and solder joints by creating homogeneous electrical contact between the functional devices and their circuits. Additionally, the repairability of E‐LIG adds another layer of value, damaged circuits can be restored on demand through relasing, enhancing durability in environments prone to mechanical or thermal stress. Our approach resolves circuit traces as small as 50 µm with excellent electrical properties, meeting the resolution requirements for advanced electronic applications. The seamless integration of conductive vias through LIG graphitization further simplifies the development of double‐sided electronics. Additionally, the transferability of E‐LIG traces onto flexible and transparent substrates such as silicone broadens the range of potential applications, particularly in wearable electronics and human–machine interfaces. We demonstrated the practical utility of the method through the integration of E‐LIG PCBs with various functional LIG devices, including strain sensor, pressure sensor, thin‐film heater, and soft electrothermal actuator. These applications highlight the versatility of the developed process and its potential to advance multifunctional printed electronics. In conclusion, our additive, laser‐enabled process not only simplifies the fabrication of high‐performance flexible electronics but also integrates a range of functional devices seamlessly onto various substrates. Future work will focus on expanding the process for multilayer PCB and 3D circuits, increasing integration density and circuit complexity. Additionally, improving the E‐LIG process with techniques like brush and electroless plating could provide automated control, while scaling the process through roll‐to‐roll manufacturing (Figure S11, Supporting Information), and exploring eco‐friendly substrates will be essential for its industrial viability.

## 4 Experimental Section

### Materials and Tools

Patterning of the porous graphene elements was achieved by direct irradiation of a commercial polyimide (PI) Kapton film that had a thickness of 127 µm (McMaster‐Carr, IL, USA) using a CO_2_ laser with a wavelength of 10.6 µm (GCC Spirit GLS‐30v). E‐LIG was also successfully formed on PI of various thicknesses (25, 50 µm) which can be found reported in Note S2 and Figure S12 (Supporting Information). This same PI sheet was used to create the solder paste mask for the PCBs. Low‐temperature solder paste used for reflowing was Chip Quik NC191LTA10 (ChipQuik, ON, Canada). The elastomer used for coating and transfer was PDMS (Dow Corning Corp., Sylgard 184) which was mixed at a ratio of 10 (base):1 (curing agent) and allowed to cure in the oven at various temperatures. For coating purposes, it was cured at 60 °C for 2 h.

### Circuit Design and LIG Patterning

The PCB was designed in Fusion 360 after assembling the components available in the library in an electronic design schematic. Once formed, the design was exported as a PNG to be formatted in Adobe Illustrator which allowed for the addition of tabs. Tabs consisted of small rectangular connections made between certain traces to allow current to flow through all areas that were to be plated as well as larger rectangles in the outer regions of the design where the connection from the power source to the LIG pattern would be established through wires. The design was then exported to CorelDraw where vias were added as a different layer to allow the vias to form under different laser speed and power settings. Vias consisted of small circles, usually 0.25 mm in diameter, to be lasered at a high speed and power to form a hole in the PI. This allowed the traces on one side of the PI to form a connection to traces on the other side because the lasering process would form LIG on the interior of the hole formed in the PI. The laser settings that were optimized for the patterning were 8% output power (maximum output power 30 W), 10% movement speed, and 1500 pulses in.^−1^.

### Copper Electroplating

Preparation of the LIG pattern for copper electroplating required copper tape, solder, PI tape, and thin wires. The wires were stripped about ⅛ in. from each end and individually soldered onto a piece of ¼ in. copper tape. The copper tape was then cut so each piece was attached to one wire, and these pieces were then attached to the tabs of the LIG pattern. The PI tape was placed on top of areas where copper tape and solder were exposed to discourage copper plating on those areas and pushed toward the LIG pattern instead. LIG patterns were electroplated using a standard copper sulfate bath consisting of 710 mL of distilled water, 14.5 g of copper sulfate, 11 g of citric acid, a pinch of polyethylene glycol, and a pinch of iodized salt. All components were allowed to mix on a hot plate solution (60 °C), to promote dissolving of particles. The solution was contained in a plastic beaker with a 1/8 in. × 1 in. × 6 in. copper bar anode immersed inside the bath. A plating power supply was used to apply a current for electroplating. A small air pump was used inside the bath to add agitation to the solution removing bubbles from potentially sticking to the surface and causing nonuniform plating. All plated samples were rinsed with isopropyl alcohol after removal from the plating bath. Once dry, the tabs were removed using a medical scalpel by hand; this process could be replicated using the tab design to laser out those portions automatically. The sample was given a final rinse and left to dry before the population.

### System‐Level Integration

All fabricated circuit boards were based on Atmel SAMD11 microcontroller model ATSAMD11C14A‐SSUTD (Microchip Technology, AZ, USA). These boards were initially programmed using the Atmel‐ICE development tool and Atmel Studio Software for the bootloader. Later, Arduino IDE was used to flash various programs. After using a mask to apply solder paste to the plated circuit board, SMD components including LEDs, resistors, voltage regulators, capacitors, nMOSFET transistors, and the SAMD11 microcontroller were placed on their respective pads. The populated circuit board was then placed in an oven at 135 °C for ≈5 min to allow the solder paste to melt and for the components to settle into place. Afterward, the sample was taken out of the oven and left to cool before adding any external components such as USB plugs using Z‐tape. The sample was tested to see if there were any issues to be debugged, and if not, the board was coated in a thin layer of PDMS and left to cure in the oven at 85 °C for at least 20 min.

### Characterization

The microstructures LIG and Cu–LIG were analyzed using an optical microscope (Lynx Evo Projection Microscope, Vision Engineering) and scanning electron microscope (FlexSEM 1000, Hitachi and Quattro ESEM, Thermo Scientific). The confocal Raman microscope spectrum (Apyron, WITec) was acquired with a laser wavelength of 532 nm. All resistance measurements for electrical characterization were captured using data logging and acquisition systems (USB‐231 USB DAQ, Digilent, WA, USA).

## Conflict of Interest

The authors declare no conflict of interest.

## Supporting information



Supporting Information

Supplemental Video 1

Supplemental Video 2

Supplemental Video 3

Supplemental Video 4

Supplemental Video 5

Supplemental Video 6

Supplemental Video 7

Supplemental Video 8

Supplemental Video 9

Supplemental Video 10

Supplemental Video 11

Supplemental Video 12

## Data Availability

The data that support the findings of this study are available from the corresponding author upon reasonable request.
