# Supplementary material for: Laser‐Enabled Fabrication of Flexible Printed Electronics with Integrated Functional Devices
Source: Adv Sci (Weinh). 2025 Mar 4;12(20):2415272. doi: 10.1002/advs.202415272 (PMC12120706; doi:10.1002/advs.202415272)
Supplement: Supplementary file 1 — Supporting Information [file ADVS-12-2415272-s006.docx]

Supporting Information

Laser-Enabled Fabrication of Flexible Printed Electronics with Integrated Functional Devices

Wedyan Babatain*, Christine Park, Hiroshi Ishii and Neil Gershenfeld

W. Babatain, C. Park, H. Ishii

Media Lab

Massachusetts Institute of Technology

Cambridge, MA, USA
E-mail: [wedyan@mit.edu](mailto:wedyan@mit.edu)

N. Gershenfeld
Center for Bits and Atoms

Massachusetts Institute of Technology

Cambridge, MA, USA

W. Babatain, C. Park, H. Ishii

Department of Electrical Engineering and Computer Science

Massachusetts Institute of Technology

Cambridge, MA, USA

Keywords: Printed Electronics, Flexible Electronics, Digital Fabrication, Laser Writing, Additive Manufacturing


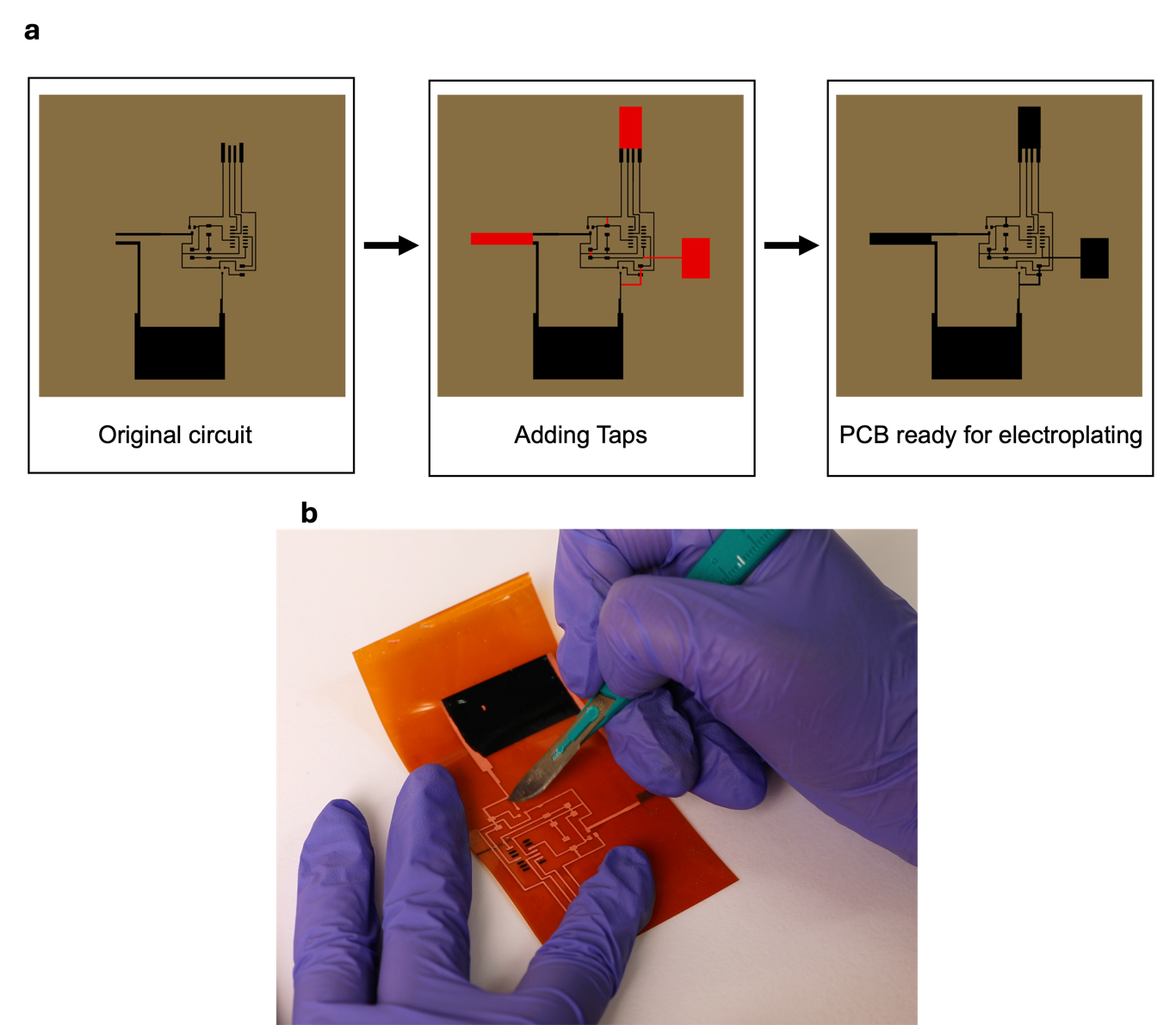


**Figure S1.** Tabs Formation Design for E-LIG Circuit Fabrication. a) Step-by-step illustration of the tab formation process for selective electroplating. The first panel shows the original circuit design. In the second panel, red sections indicate the addition of conductive tabs, which serve as temporary pathways to ensure uniform copper deposition during electroplating. In the third panel, the finalized circuit design with tabs is shown, ready for the electroplating process. (b) Manual removal of the conductive tabs post-plating, using a blade, to eliminate any undesired connection between segments. This step ensures that only the intended circuit pathways remain connected.


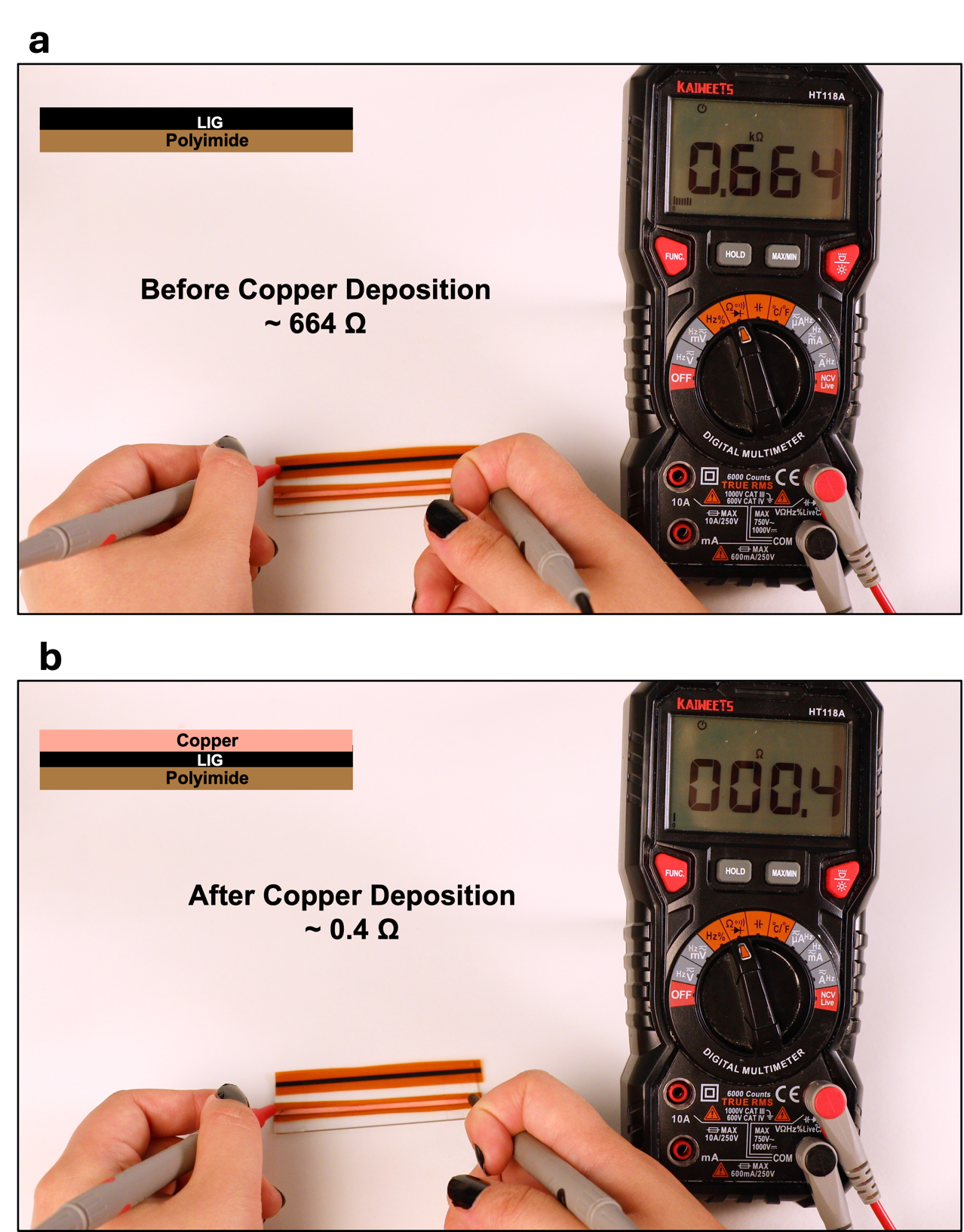


**Figure S2.** Resistance Reduction of LIG Trace Before and After Copper Electrodeposition. a) Measurement of the initial resistance of the LIG trace on a polyimide substrate before copper electroplating, showing approximately 664 Ω. The LIG layer alone provides moderate conductivity suitable for initial circuit formation but not sufficient for high-performance applications. b) Measurement of the same trace after copper deposition, with the resistance significantly reduced to approximately 0.4 Ω. The copper layer on top of the LIG dramatically enhances conductivity, demonstrating the effectiveness of the E-LIG process in achieving low-resistance traces suitable for practical electronic applications.


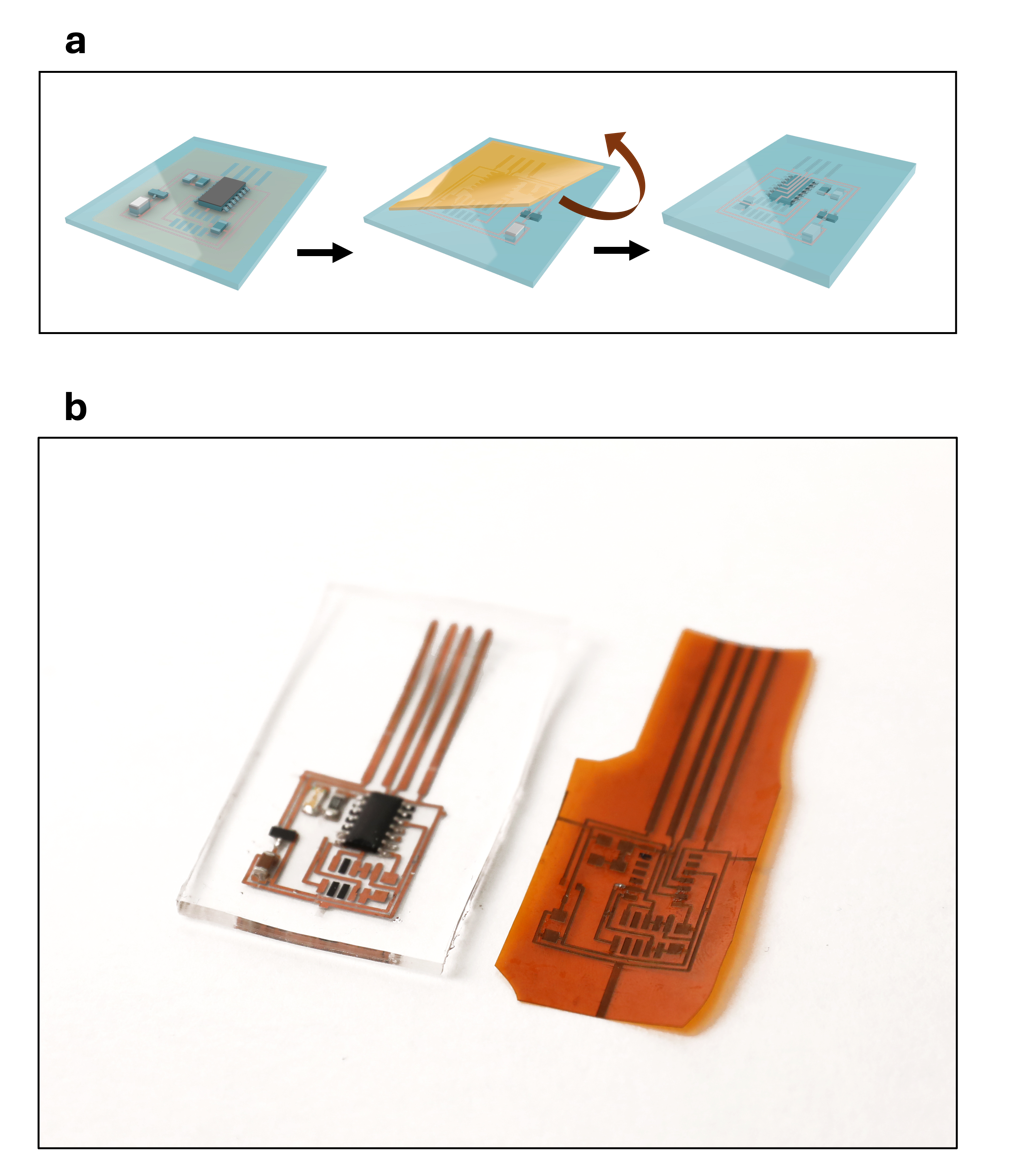


**Figure S3.** Transfer Process of E-LIG Circuit onto PDMS. a) Illustration of the transfer process where the LIG circuit, complete with copper plating and surface-mount components, is embedded onto a PDMS substrate. The polyimide (PI) layer is peeled away after curing, leaving the E-LIG traces, and mounted components fully embedded within the flexible PDMS substrate. (b) Photograph showing the result of the transfer process. The PDMS substrate (left) retains the entire circuit, including the E-LIG traces and components, while the PI layer (right) has been successfully stripped out of all components, demonstrating the effectiveness of the transfer process.


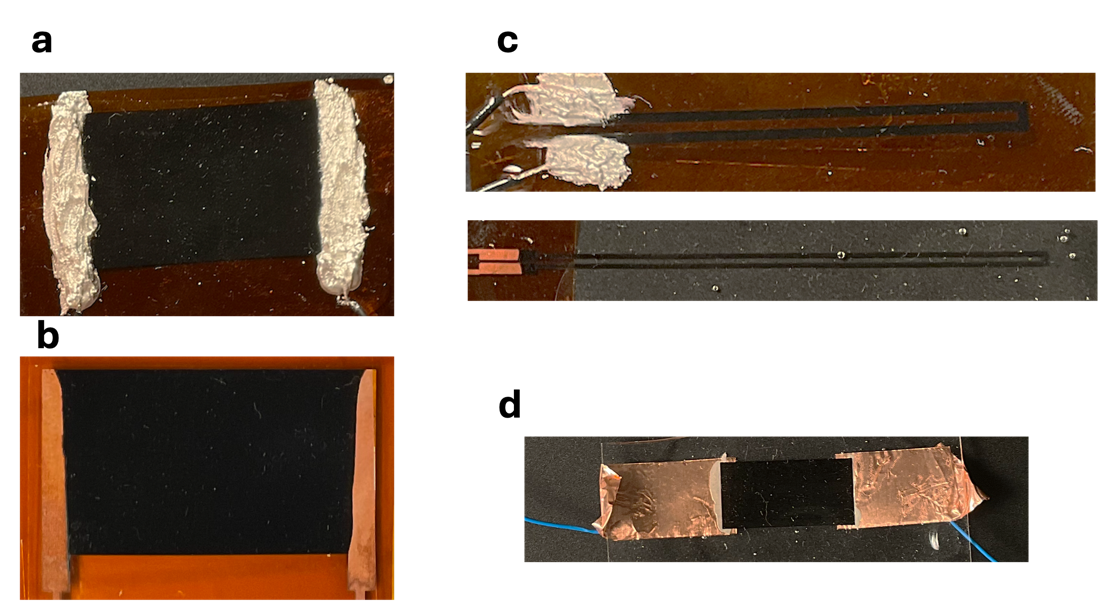


**Figure S4.** Transfer Process of E-LIG Circuit onto PDMS. a) Illustration of the transfer process where the LIG circuit, complete with copper plating and surface-mount components, is embedded onto a PDMS substrate. The polyimide (PI) layer is peeled away after curing, leaving the E-LIG traces, and mounted components fully embedded within the flexible PDMS substrate. (b) Photograph showing the result of the transfer process. The PDMS substrate (left) retains the entire circuit, including the E-LIG traces and components, while the PI layer (right) has been successfully stripped out of all components, demonstrating the effectiveness of the transfer process.


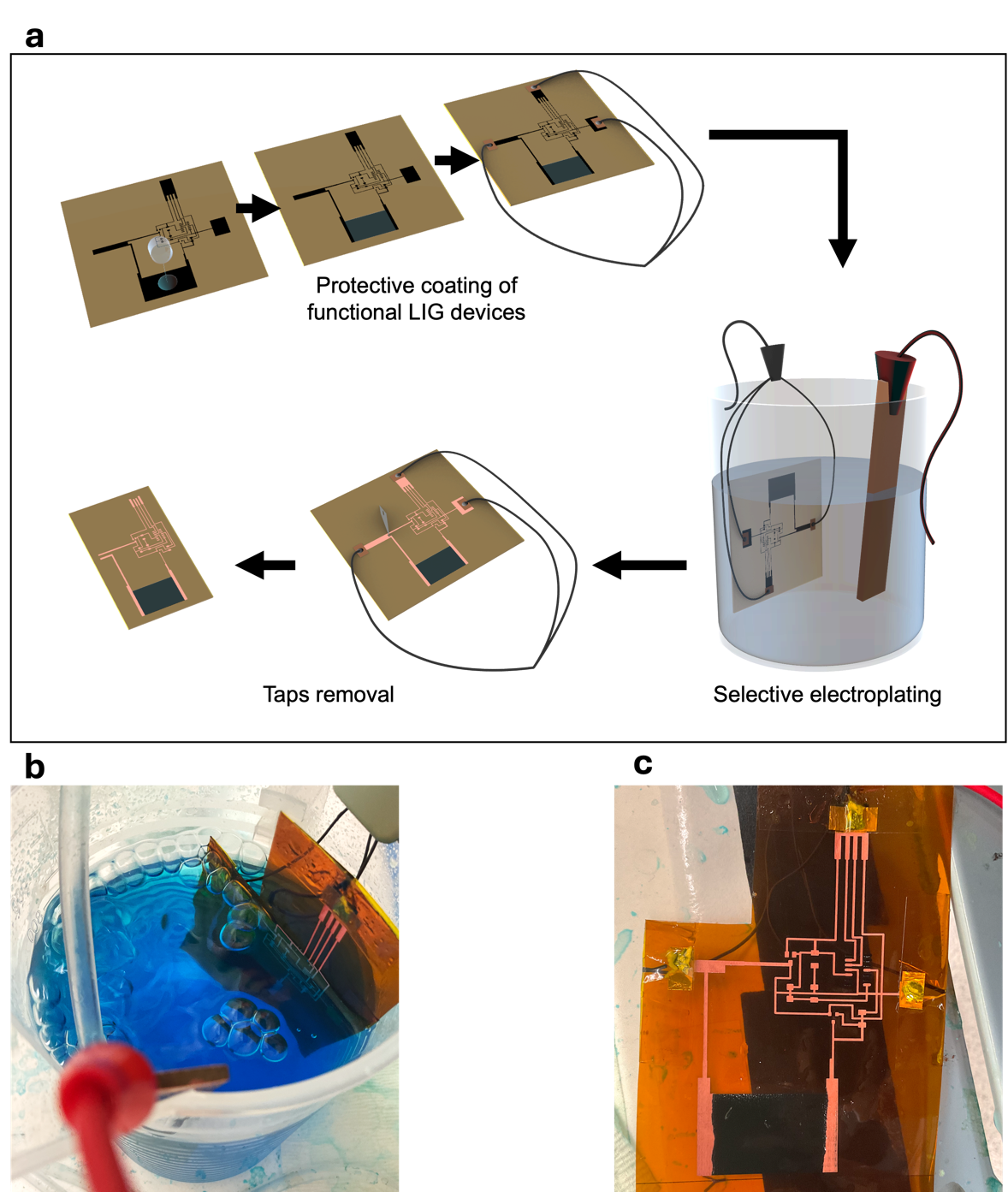


**Figure S5.** Selective Electroplating Process for Integrated E- Circuit Fabrication. This figure illustrates the selective electroplating technique that enables the copper plating of conductive traces while preserving the functional LIG-based regions, such as heaters, without additional plating. a) The process begins with a protective coating applied to the functional LIG areas, ensuring these regions remain graphitized during electroplating. The coated circuit is then immersed in a copper sulfate solution for selective electroplating. Once the electroplating is complete, tabs used for grounding are removed. b) Photograph showing the selective electroplating in action, where the coated functional area resists copper deposition while the exposed circuit traces undergo electroplating. c) Final result of a selectively electroplated circuit with copper-plated conductive paths and LIG functional regions, demonstrating the ability to combine high-conductivity pathways with functional LIG elements on a single substrate. This approach provides flexibility in integrating diverse functionalities within the same circuit.


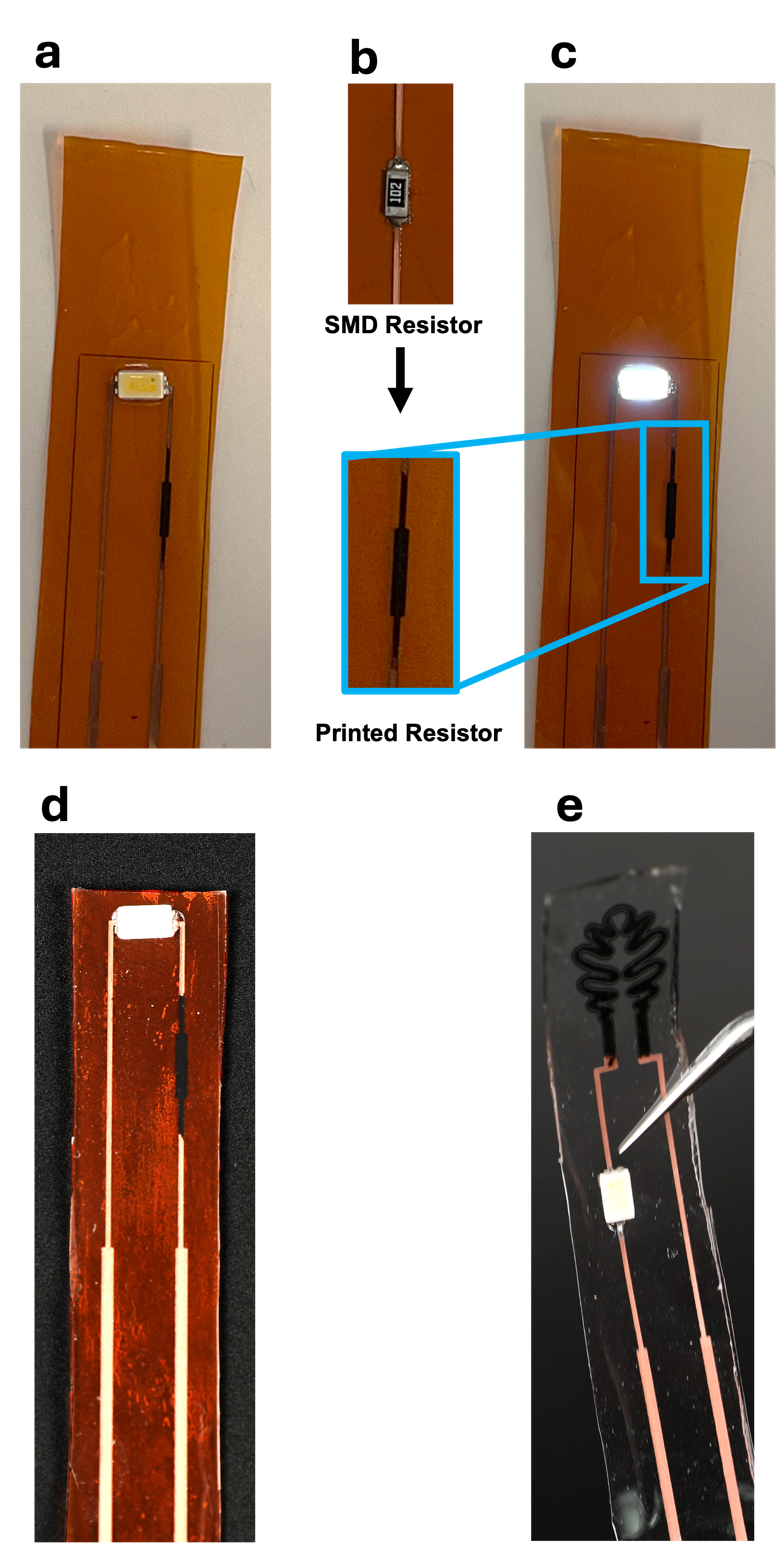


**Figure S6.** Selective Plating for Tunable E-LIG Circuit Elements and Integrated Control. This figure demonstrates the selective electroplating approach that enables the integration of both highly conductive and resistive printed components in a single circuit. a) Initial circuit layout with an unplated printed resistor serving as the current-limiting resistor for the LED, eliminating the need for an external SMD resistor. b) Comparison between an SMD resistor and the printed resistor within the circuit. The printed resistor achieves tunable resistance by avoiding copper plating, while conductive traces are fully plated to establish contact with the LED and power source. c) Fully assembled circuit with the LED turned on, showcasing the functionality of the printed resistor in place of an SMD component. d) Close-up view of the plated and unplated sections, highlighting the tunable conductivity. e) Demonstration of LED intensity control via an integrated resistive pressure sensor, allowing for real-time modulation of brightness. This approach shows the versatility of selective electroplating for creating multifunctional circuits with both passive and active elements seamlessly integrated.


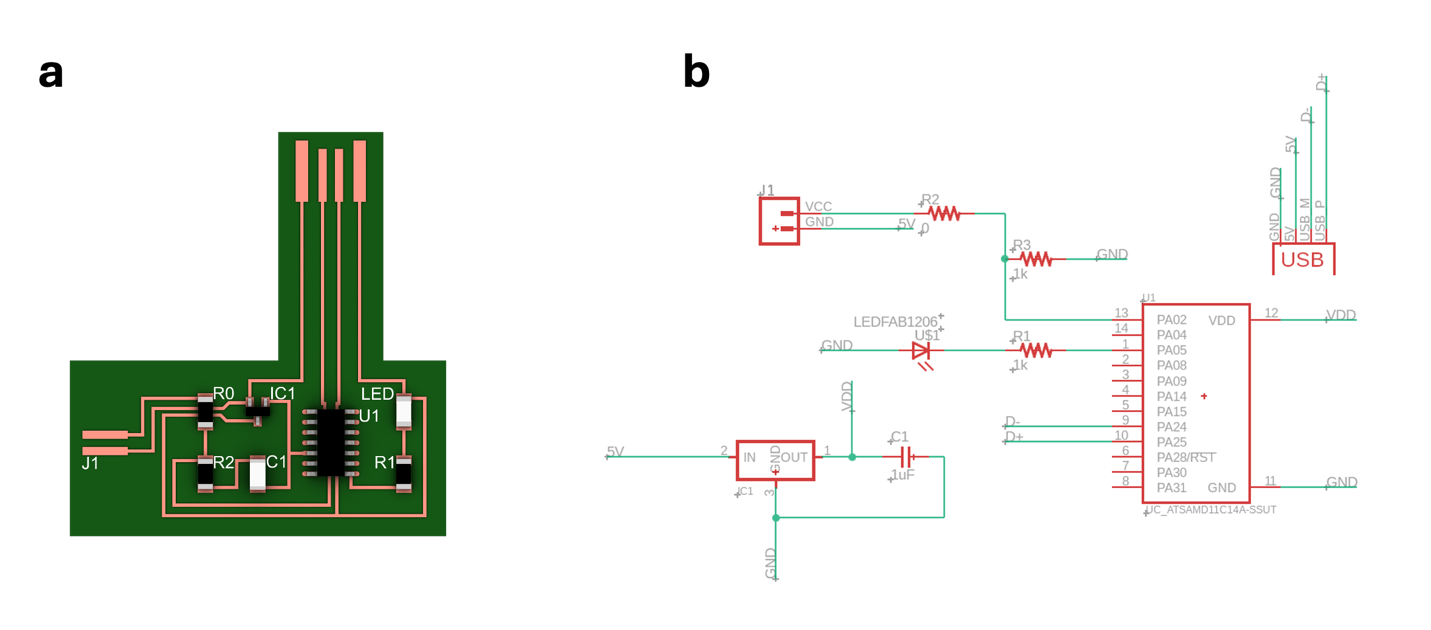


**Figure S7.** Design Layout and schematic of Flexible Sensor Circuit. a) 3D design layout of the flexible sensor interface circuit, showcasing the arrangement of various components on the circuit board. b) Circuit schematic illustrating the electrical connections within the flexible sensor circuit components

**Table S1.** Component List for the Sensor Interface Circuit

| **Components** | **Value & Series Number** | **Description** |
| --- | --- | --- |
| C1 | 1 µF | 1206 Capacitor |
| IC1 | LM3480IM3-3.3/NOPB | 3.3V Voltage Regulator_SOT23 |
| J1 | Pad connector | Sensor Connector |
| LED | SML-LX1206IC-TR | 1206 LED |
| R0 | 0 Ω | 1206 Resistor |
| R1 | 1 kΩ | 1206 Resistor |
| R2 | 1 kΩ | 1206 Resistor |
| U1 | ATSAMD11C14A-SSUT | Microcontroller |
| USB | USB | USB connector |


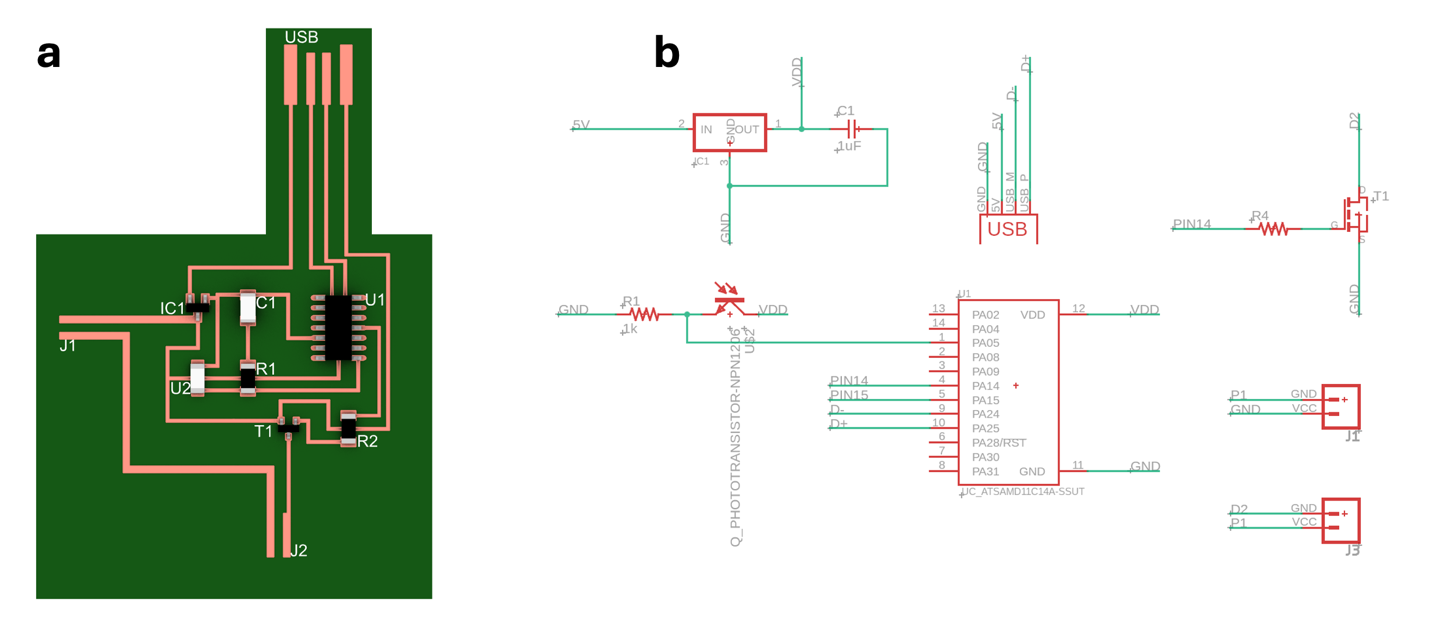


**Figure S8.** Design Layout and schematic of Actuator Control Circuit. a) 3D design layout of the actuator circuit, showcasing the arrangement of various components on the circuit board. b) Circuit schematic illustrating the electrical connections within the actuator circuit components

**Table S2.** Component List for the Actuator Control Circuit

| **Components** | **Value & Series Number** | **Description** |
| --- | --- | --- |
| C1 | 1 µF | 1206 Capacitor |
| IC1 | LM3480IM3-3.3/NOPB | 3.3V Voltage Regulator_SOT23 |
| J1 | Pad connector | Power Connector |
| J2 | Pad connector | Actuator Connector |
| R1 | 1 kΩ | 1206 Resistor |
| R2 | 100 Ω | 1206 Resistor |
| T1 | SI2336DS-T1-GE3 | N-CH MOSFET_SOT23 |
| U1 | ATSAMD11C14A-SSUT | Microcontroller |
| U2 | PT15-21C/TR8 | 1206 Phototransistor_NPN |
| USB | USB | USB connector |


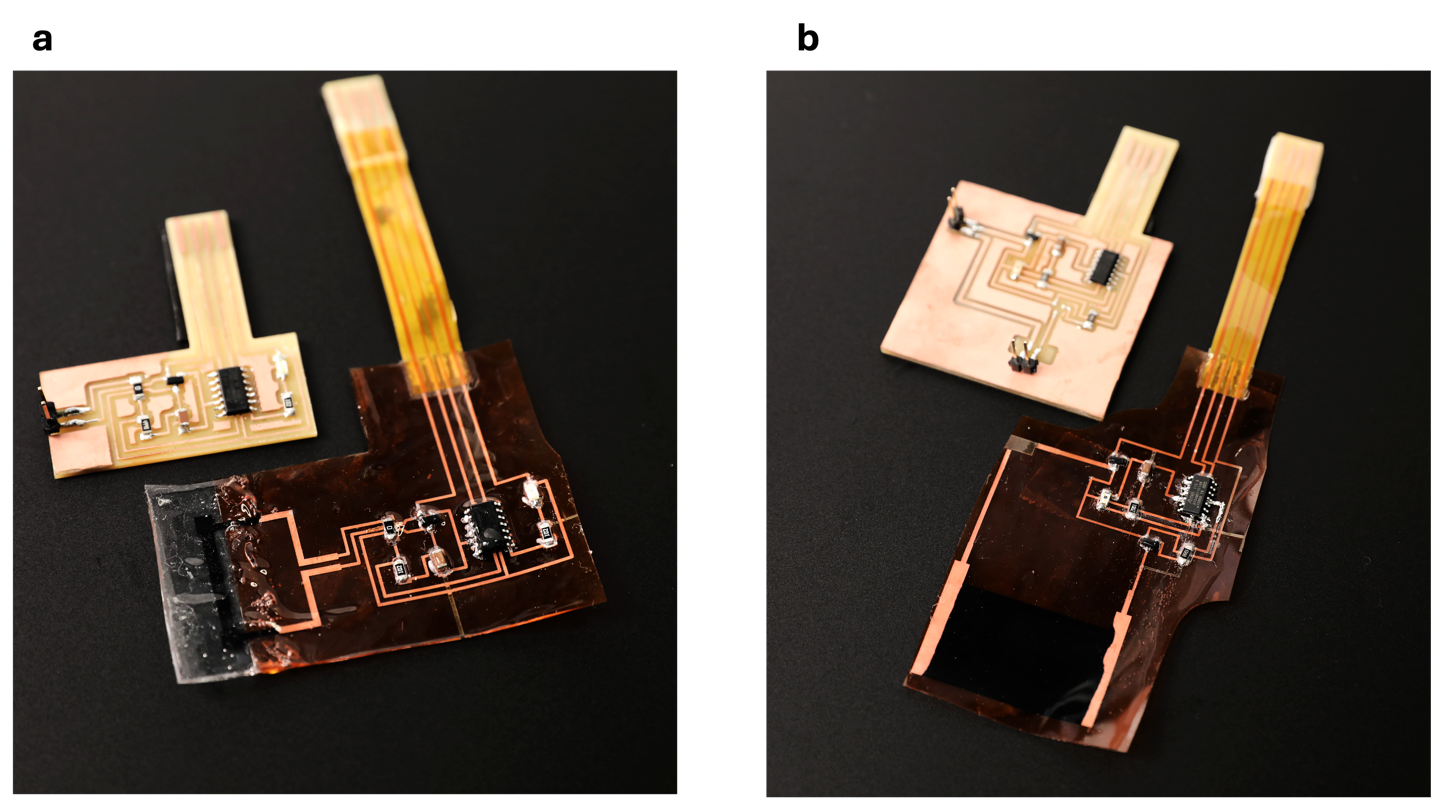


**Figure S9.** Comparison of Flexible and Rigid Versions of the Same Circuit Fabricated with E-LIG Method. a) Pressure sensor interface circuit. b) Heater control circuit.


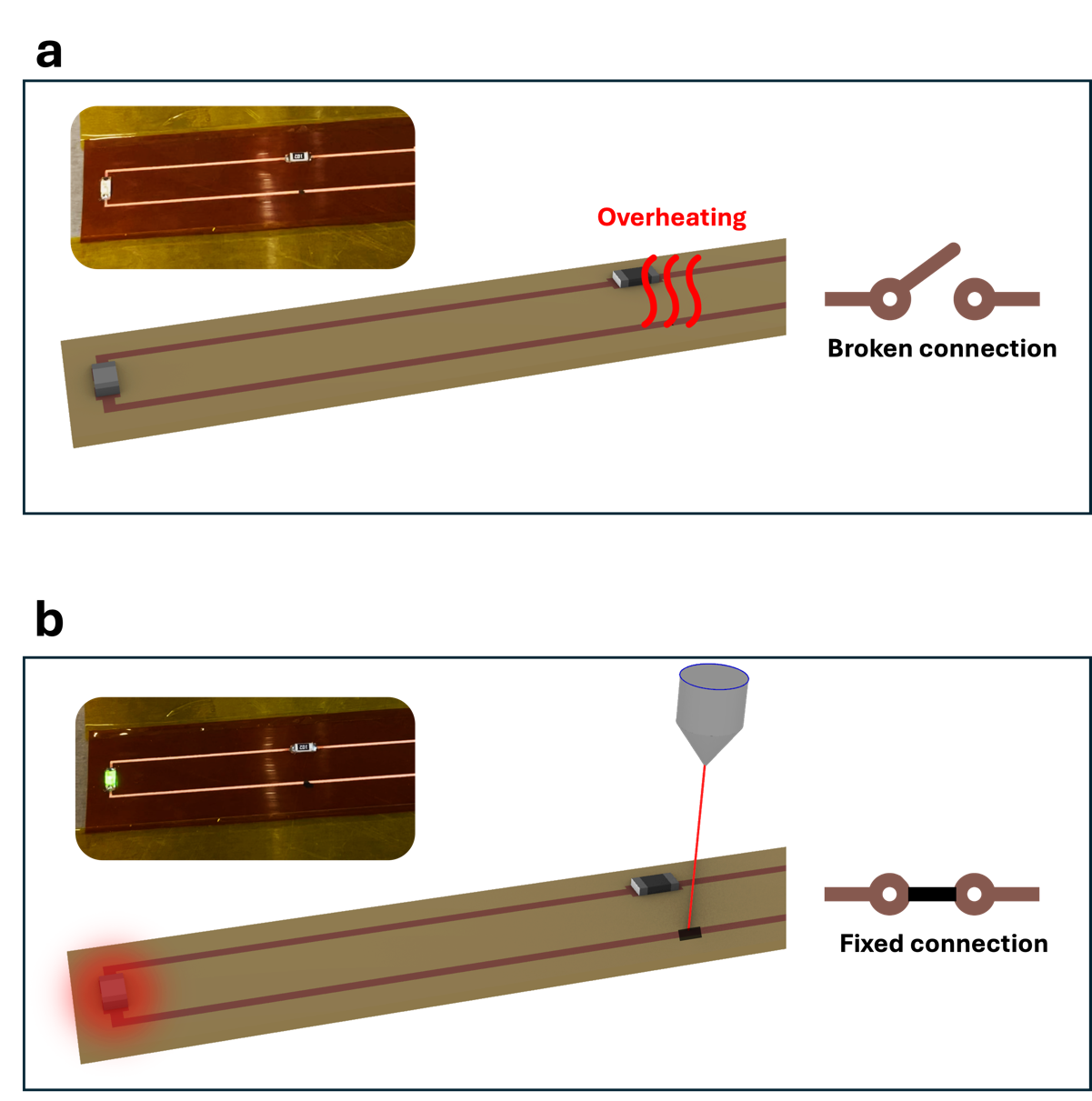


**Figure S10.** On-Demand Repairability of E-LIG Circuit Connections through Laser Re-graphitization. a) Illustration of a circuit segment where overheating could lead to a broken connection, shown in the inset image. b) Demonstration of the repair process: by re-lasing the affected area, the carbon-rich substrate provides a continuous source for graphene induction, effectively restoring the conductive pathway. The inset shows the re-established connection with the LED turning on, confirming successful repair. This process shows E-LIG method's capability for on-demand circuit restoration, extending the lifespan of flexible electronic devices in challenging environments.


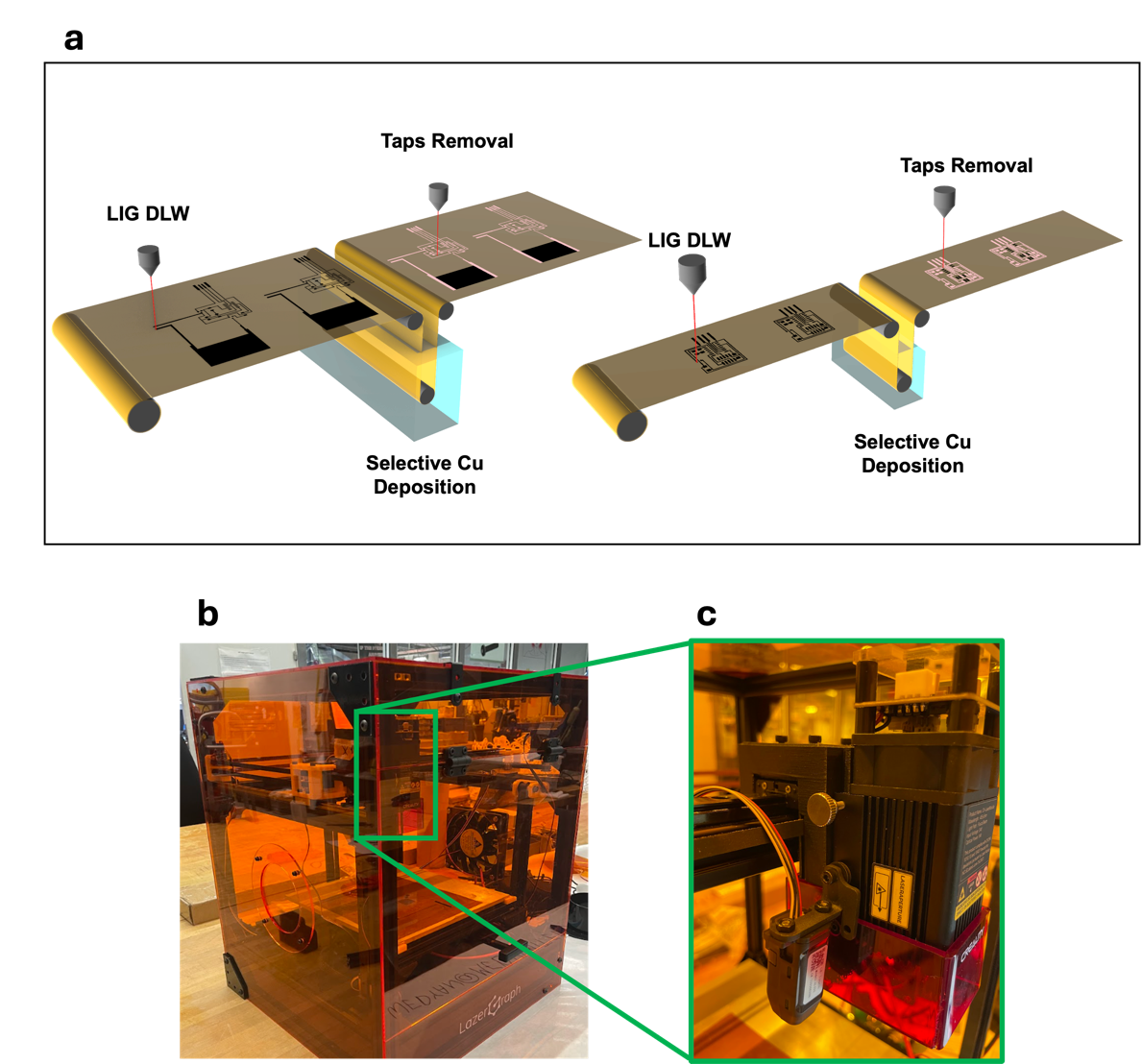


**Figure S11.** Scalability and Versatility of the E-LIG Fabrication Method. a) Illustration of the envisioned roll-to-roll manufacturing process for E-LIG fabrication, demonstrating the scalability of this technique. The process begins with direct laser writing (DLW) of the LIG pattern, followed by selective copper deposition. Automated removal of tabs completes the process, yielding conductive and functional circuit elements efficiently. (b) A custom-built, low-cost laser diode system, showcasing the adaptability of the E-LIG process to different laser types. (c) Close-up of the $100, 450 nm laser diode used in the custom system. This laser successfully supports E-LIG fabrication, highlighting the method's versatility beyond conventional CO2 lasers. E-LIG is applicable for a variety of laser configurations, supporting industrial viability.

**Note S1: Envisioned roll-to-roll manufacturing process of E-LIG**

In the envisioned roll-to-roll manufacturing process is depcited in Figure S11a, establishing electrical contact becomes impoertant.We propose two feasible approaches. The first is a probe-based contact tool where a probe with multiple small contact points (high desnity) would be used to gently press onto the substrate, establishing electrical connections across the patterned area, including flying pads and disconnected sections. This probe would initiate the plating process and then lift off once plating is completed. Alternatively, electroless plating can be utilized, where only the laser treated area, the LIG patterns are selectively plated without the need for direct electrical contact. This method works well with the envisioned roll-to-roll process and ensures efficient aconivent plating nd .Both approaches are scalable and compatible with the roll-to-roll process, ensuring the feasibility of E-LIG fabrication in large-scale manufacturing.

**Comparison of the developed method with other Flexible PCB Manufacturing Methods**

Table S3 below details the advantages and disadvantages of different PCB manufacturing method including the developed E-LIG method, highlighting its advantages and disadvantages of when it compares with other fabrication methods.

**1. Screen Printing**

Screen printing involves the transfer of conductive inks and other such functional materials onto a substrate using a mesh screen and stencils to form the desired circuit pattern. This method is useful for large-scale productions of various electronics, and it is versatile in allowing the use of different materials and substrates to form flexible and rigid electronics. However, this method struggles with resolution and complex electronics that have multiple layers and/or require precision for fine features^[1]^.

**2. Inkjet Printing**

Inkjet printing can be distinguished into two main types: continuous inkjet (CIJ) and drop-on-demand inkjet (DOD). Continuous inkjet printing relies on a continuous stream of droplets of a chosen material to be deposited onto a substrate after passing through charging plates by being guided by an electric field. The continuous stream of droplets requires the unused droplets to be collected separately and recirculated, and this issue is addressed by drop-on-demand inkjet printing which only deposits the droplets on demand. Both methods are compatible with various materials and substrates and are scalable with the use of multiple printheads. Drop-on-demand inkjet printing provides further improvements by being more efficient than continuous inkjet printing and reducing the risk of contamination through the recirculation of droplets, but this method suffers with clogging issues when the printhead is idle^[2]^.

**3. Direct Ink Writing**

Direct ink writing is a method that, similar to screen printing, involves depositing a chosen material (often solder pastes, conductive inks, and other functional materials) onto a substrate layer by layer through a nozzle. This method is highly compatible with methods like screen printing by being able to utilize screen-printable inks and pastes, and it is versatile with various materials and substrates. Direct ink writing is also more precise which allows for higher-resolution electronics that involve detailed features. On the other hand, due to forming patterns feature by feature, this method is slower than methods such as screen printing, and it relies on ink cartridges that often need to be replaced ^[1]^. Moreover, E-LIG can be easily transferred to other substrates. The main advantages of transferring E-LIG over direct ink writing of silver paste is the complexity of each method and the ease of integrating LIG devices such as heaters and sensors to the main circuit. Direct ink writing of silver paste requires a specialized 3D printer and nozzle to extrude the paste with the precision needed to form high-resolution circuit patterns whereas the pattern desired for an E-LIG device can be manipulated in CAD software to match the parameters for any CO2 laser. Furthermore, LIG devices can be integrated within the initial circuit design while still allowing key components of the pattern to be copper electroplated, and the transfer of the design is made seamless by manipulating the amount of elastomer on different portions of the circuit when needed. This feature proves to be an advantage over the risks that can occur with trying to integrate LIG devices while curing the silver paste involved with direct ink writing which usually involves a thermal or chemical reaction. Additionally, the mismatch between the two materials at the LIG device and silver past electrical contact interface could introduce challenges such as increased contact resistance and mechanical mismatch^[3,4]^.

**Table S3.** Comparison of Flexible PCB Manufacturing Methods for Conductive and Functional Device Integration

| Method | Resolution | Conductivity | Via/Multilayer Capability | Process Complexity | Material Compatibility | Cost Efficiency | Scalability | Environmental Impact | Functional Integration of Other Devices | Ref. |
| --- | --- | --- | --- | --- | --- | --- | --- | --- | --- | --- |
| E-LIG  **(This work)** | ~50 µm | Very High | Enabled (Laser-formed VIAS + Cu plating) | Simple  (DLW + Electroplating) | Flexible, Transparent (PI, PDMS, other elastomers) | High (low material waste, maskless) | High potential (roll-to-roll, large area) | Low waste, maskless process | High, seamless integration of LIG-based sensors/actuators | This work |
| Current Commercial Flexible PCB Methods | 50-75 µm | Very High | Enabled, (multiple steps needed for vias) | Multi-steps (Photolithography, multiple chemical baths, drilling) | Polyimide (PI), PET | Moderate to High | Very High (optimized for roll-to-roll) | Moderate to High (complex processes, chemicals) | Moderate, additional steps for integration | PCBWay ^[5]^ |
| Inkjet Printing | 20–50 µm | Moderate | Limited (via hole filling challenging) | Moderate (ink formulation + curing) | Flexible substrates (PI, PET) | Moderate (Requires custom ink preparation, post-processing) | High | Moderate (inks waste generation from ink formulation | Moderate, limited material interaction | ^[6]^ |
| 3D Printing (Direct Writing) | ~20 µm | Moderate (conductive polymers) | Limited | Moderate (precise extrusion control) | Flexible, stretchable substrates | Moderate (Specialized printers and materials) | Moderate (slow speeds) | Low waste | High (introducing other inks, multi-material printing) | ^[7]^ |
| Screen Printing | ~30-100 µm | Moderate (carbon or silver inks) | Limited | Moderate (printing + curing) | Flexible, stretchable substrates | Moderate (simple, cost-effective for bulk) | High | Low waste | Moderate, limited to passive components | ^[8]^ |
| Spray Coating | ~100 µm | Moderate (conductive inks) | Limited | Simple, requires masking | Flexible, stretchable substrates | High (low-cost method for large areas) | High | Moderate (waste from solvent) | Moderate, suitable for basic printed components | ^[9]^ |

**Note S2: LIG and E-LIG formation on polyimide (PI) films of varying thicknesses**

As reported, the PI film was used in this study is 127 µm in thickness. It is imprtant to note that this is thicker than the common industry standard PCB films of 25 µm or 12.5 µm. The initial choice of thicker PI film was deliberate to ensure that after laser conversion of PI to laser-induced graphene (LIG), a sufficient PI layer remains underneath the LIG structure to support ease of handling during fabrication and characterization and avoid burning through the PI sheet completely due to the high energy density required for graphene formation. It is also worthy to mention that the majority of reported LIG in the literature utilizes such thickness for the same main reason ^[10]^. Nonetheless, this does not mean that we are limited by this thickness. Some studies in the literature have successfully formed LIG on thinner PI films such as 50 µm^[11]^ and 60 µm ^[12]^ and even down to 12 µm ^[13]^ by a recent study that mitigated the burn-through issue using auxiliary cooling methods to prevent damage during the laser induction process. We have expanded our experiments to include LIG formation on thinner PI films to demonstrate the versatility of our method. Specifically, we successfully generated LIG on a 50 µm PI sheet (from DuPont) and a 25 µm polyimide tape (commonly referred to as Kapton tape). These thinner films are widely available, accessible, and commonly used in flexible electronics applications. Figure S12 shows photographs of LIG and E-LIG electrodes generated on 25 µm, 50 µm, and 127 µm PI films, along with their corresponding measured resistances before and after copper plating. In Figure S12a, LIG and E-LIG electrodes are generated on a 25 µm commodity PI tape (Figure S12d), demonstrating the feasibility of forming LIG on thin, commercially available substrates. Figures S12b and S12c show similar results on 50 µm and 127 µm PI sheets, respectively. Notably, across all thicknesses, the copper plating process significantly reduces the resistance of the LIG traces to approximately 0.2 Ω, regardless of the initial resistance of the LIG seed layer.

Below are our findings comparing the initial resistance of a 2 mm × 20 mm LIG electrode across different PI thicknesses, as well as the voltage and time required for their subsequent copper plating:

**Table S4**: Resistance and Plating Conditions of LIG formed on Different PI Thicknesses

| **PI Thickness (µm)** | **R _LIG_ (Ω)** | **Plating Voltage (V)** | **Plating Time (min)** | **R _Cu_LIG_ (Ω)** |
| --- | --- | --- | --- | --- |
| 25 µm (PI tape) | 1623 | 0.5 | 40 | 0.2 |
| 50 µm (PI sheet) | 294.2 | 0.5 | 20 | 0.2 |
| 127 µm (PI sheet) | 193.2 | 0.5 | 12 | 0.2 |

As shown in table S4, all samples achieved the same final low resistance of 0.2 Ω after plating. However, thinner films required more time to achieve the same plating results due to the higher initial resistance of the LIG seed layer. The higher initial resistance is expected and is a direct result of the thinner LIG layer produced on thinner PI substrates. This increase in time required to plate less conductive thin LIG seed layer can be mitigated by tuning other plating parameters such as solution concentration, plating voltage, and current to achieve uniform and efficient copper deposition on different substrate thicknesses.


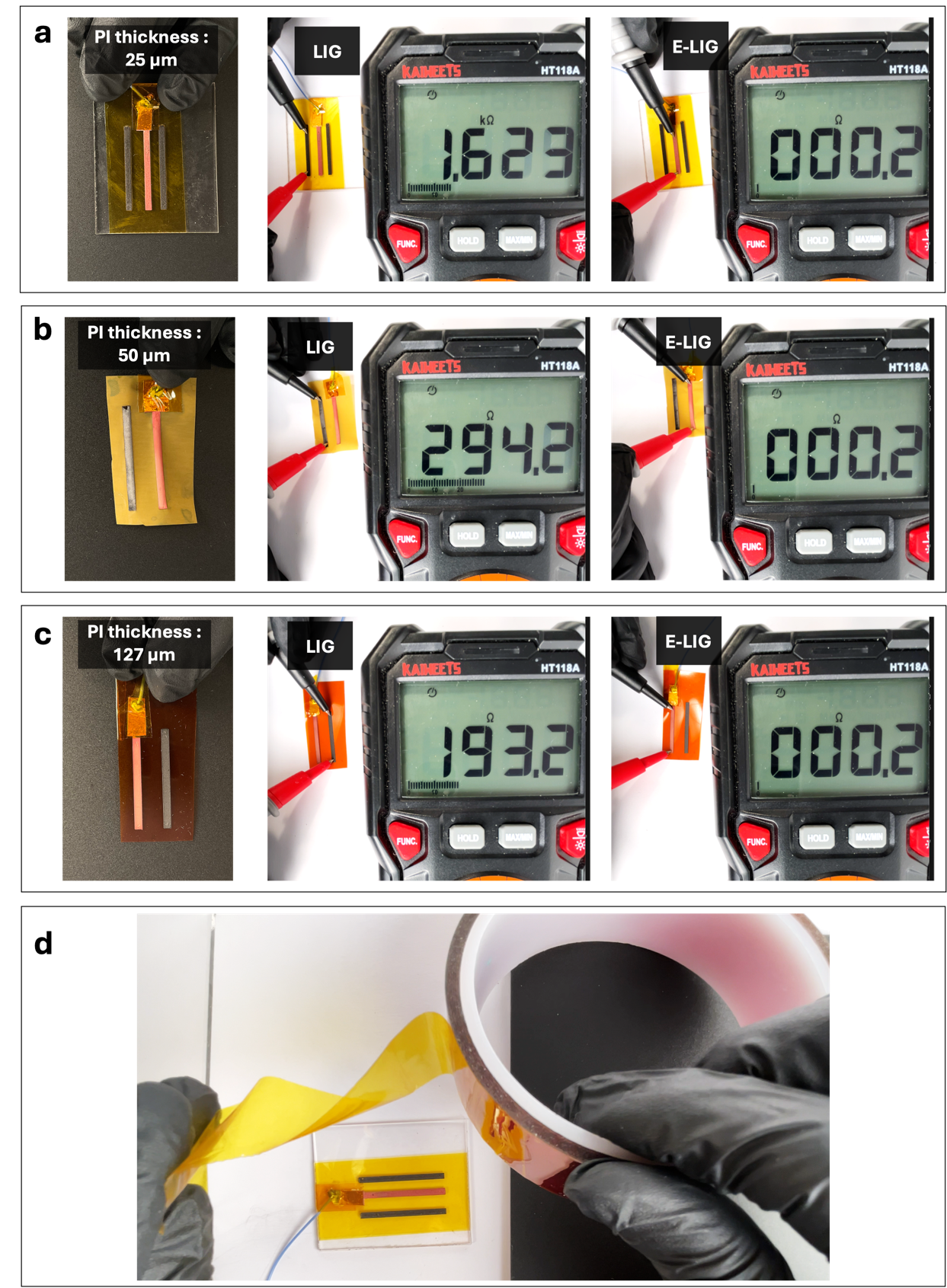


**Figure S12.** LIG formation and LIG copper plating on polyimide (PI) films of varying thicknesses. (a) Photographs of LIG and E-LIG electrodes generated on 25 µm PI tape, with corresponding measured resistances before and after copper plating. (b) Photographs of LIG and E-LIG electrodes generated on 50 µm PI sheet, with corresponding measured resistances. (c) Photographs of LIG and E-LIG electrodes generated on 127 µm PI sheet, with corresponding measured resistance. (d) Photo of the 25 µm commodity PI tape used to generate LIG and E-LIG electrodes.

**Note S3: Sheet resistncae meausrment and conducitivty of LIG and E-LIG**

We have measured the sheet resistance of the laser-induced graphene (LIG) seed layer and the copper plated LIG layer (cu-LIG) using four-point measurement tool. The probing current was 100 mA.We also measured the corresponding thickness of these layers in order to accurately calculate the conductivity of both layers.

**For LIG**

Measured Sheet resistance is of LIG $R_{s}= 31.05 (m\Omega/sq)$

Measured Thickness of LIG is $t=18 (\mu m)$

Calculating the resistivity of LIG $\rho_{LIG}$

$\rho_{LIG}=R_{s}\times t$ (1)

$\rho_{LIG}=31.05 (m\Omega/sq)\times18 (\mu m)$ (2)

$\rho_{LIG}=[31.05\times{10}^{-3}\left( \frac{\Omega}{sq} \right)]\times[18{\times10}^{-6} \left( m \right)]$ (3)

$\rho_{LIG}=5.589\times{10}^{-7}\left( \Omega\cdot m \right)$ (4)

Calculating the conductivity of LIG $\sigma_{LIG}$

$\sigma_{LIG}=\frac{1}{\rho}=\frac{1}{5.589\times{10}^{-7}}$ (5)

$\sigma_{LIG}=$1.789 $\times{10}^{6}$ (S/m) (6)

**For E-LIG / Cu-LIG**

Measured Sheet Resistance of Cu-LIG is $R_{s}= 2.67 (m\Omega/sq)$

Measured Thickness of Cu-LIG is $t=9 (\mu m)$

Calculating the resistivity of Cu-LIG $\rho_{Cu-LIG}$

$\rho=R_{s}\times t$ (7)

$\rho_{Cu-LIG}=2.67 (m\Omega/sq)\times9 (\mu m)$ (8)

$\rho_{Cu-LIG}=[2.67\times{10}^{-3}\left( \frac{\Omega}{sq} \right)]\times[9{\times10}^{-6} \left( m \right)]$ (9)

$\rho_{Cu-LIG}=2.4\times{10}^{-8}\left( \Omega\cdot m \right)$ (10)

Calculating the conductivity of Cu-LIG $\sigma_{Cu- LIG}$

$\sigma_{Cu- LIG}=\frac{1}{\rho}=\frac{1}{2.4\times{10}^{-8}}$ (11)

$\sigma_{Cu- LIG}=$4.16 $\times{10}^{7}$ (S/m) (12)

The calculation above yields conductivity of 1.789 $\times{10}^{6}$ S/m for LIG and 4.16 $\times{10}^{7}$S/m for Cu-LIG, which are consistent with the reported values in the literature for LIG ^[10,14,15]^ and copper films ^[16]^. These values indicate that the electroplating process yields high level of conductivity in the copper layer, comparable to conventional copper films used in flexible electronics.

**S4: The effect of Tabas formation technique on the resistance of seed layer for laarge-are pattern**

It is important to note that the conductivity of the seed layer affects the efficiency of copper plating, especially for large-area patterns where longer and thinner traces can lead to increased resistance, thus reducing plating efficiency. To address this concern regarding plating efficiency over large-area LIG patterns, we demonstrate a tabbing method designed to improve electrical connectivity across large patterns, thereby mitigating the challenge of high resistance, which can hinder the plating process. The tabbing method introduces temporary pseudo-traces (tabs) that establish multiple electrical contact points across the circuit during plating, thereby reducing the overall resistance of the layout.In Figure S13, we compare the same 10 cm × 10 cm LIG LED grid pattern with and without tabs. Column (a) shows the resistance measured at various points on the pattern without tabs, and column (b) shows the corresponding measurements on the same pattern with tabs added. The measurements were taken at the same points across both patterns, as highlighted by the yellow circles in Figure S13 for direct compations between the two designs, illustrating the impact of tabs on reduceing resistance to certain points.

The addition of temporary tabs significantly reduces the resistance at various points by providing shorter electrical paths to the nearest connection, which would make electroplating large-area patterns more efficient and uniform. This tabbing approach helps to overcome the challenge of high resistance over long traces by distributing the electrical contact points more evenly across the pattern. The tabs can be easily removed after plating, either manually or via laser etching, to avoid undesired connections in the final circuit.

In addition to the tabbing method, we propose two alternative strategies that can be considered to further improve the conductivity of the seed layer in future work. One approach is flash healing, which involves applying a high-energy pulse to the LIG seed layer to enhance its conductivity. This process has been shown to significantly reduce the sheet resistance of laser-induced graphene, thereby improving plating efficiency without compromising the integrity of the polyimide ^[17]^. Another promising strategy is electroless plating where prior to electroplating, a thin, uniform copper layer can be deposited selectively on the LIG seed layer using an electroless plating solution. This initial conductive layer helps to reduce the resistance of the seed layer, thereby facilitating more efficient electroplating. The electroless plating process is particularly beneficial for large-area patterns^[18,19]^. Together, these strategies offer promising avenues for future exploration to enhance the conductivity of the LIG seed layer and improve plating efficiency in large-area flexible electronics.

**
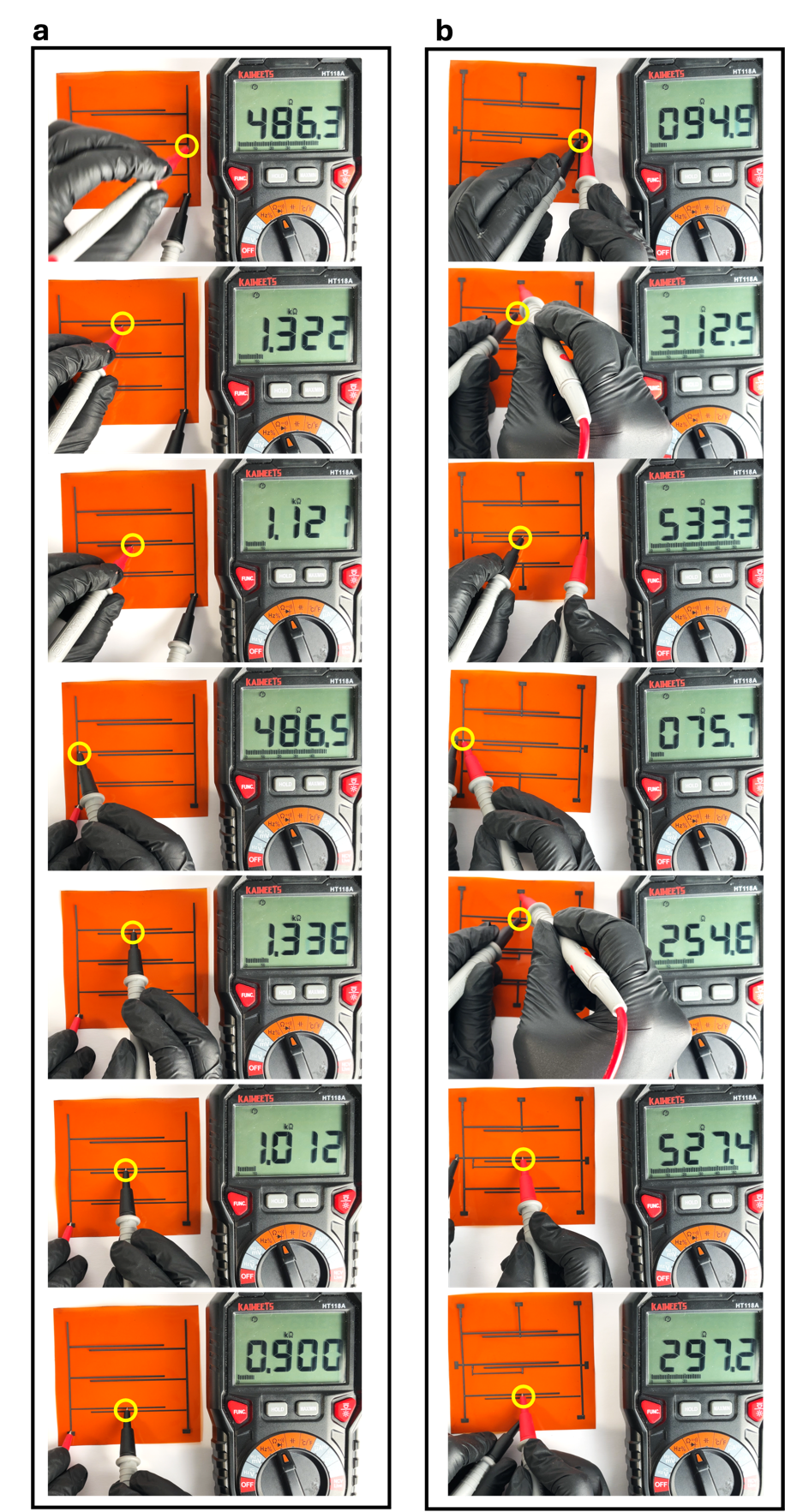
**

**Figure S13.** Comparison of 10 cm × 10 cm LIG LED grid patterns with and without temporary tabs to reduce resistance across large areas. (a) Resistance values measured at various points on the pattern without tabs. (b) Resistance values measured at the same points on the pattern with tabs added. The yellow circles highlight the measurement points. Tabs reduce resistance by shortening electrical paths, enabling efficient electroplating in large-area applications.

**Note S5 : Mechanical Testing**

In order to test the adhesion strength between the copper electroplated LIG layer (E-LIG) and the PI substrate, we conducted a series of tests to evaluate the adhesion force and mechanical robustness of the copper-plated LIG on PI film and its transferred versions on PDMS. The adhesion strength of E- LIG circuits was assessed through a series of mechanical tests, including bending test, strain test, and tape test, conducted on three different sample types. Those samples are E- LIG on polyimide (PI), E - LIG on PI with a thin PDMS coating. Transferred E-LIG on PDMS with PDMS coating on both sides. All samples had a trace width of 2 mm.

**1. Bending Test (1000 Cycles at 7.5 mm Radius):**

The bending test was conducted using a custom-built stepper motor-driven linear setup (Figure S14a, S14b and S14c). Samples were subjected to repetitive bending at a radius of 7.5 mm for 1000 cycles to test the mechanical integrity of the copper-plated LIG circuits under cyclic deformation. The unbent state of the sample is shown in Figure S14a, while the bent state during the test is shown in Figure S14b. The bending radius of 7.5 mm, as viewed from the top, is depicted in Figure S14c. After 1000 bending cycles, all three samples showed no visible cracking, delamination, or mechanical damage, as shown in Figure S14d. This result confirms that the copper-to-LIG bond and LIG-to-PI bond remain robust under repetitive bending, which is critical for flexible electronic applications.


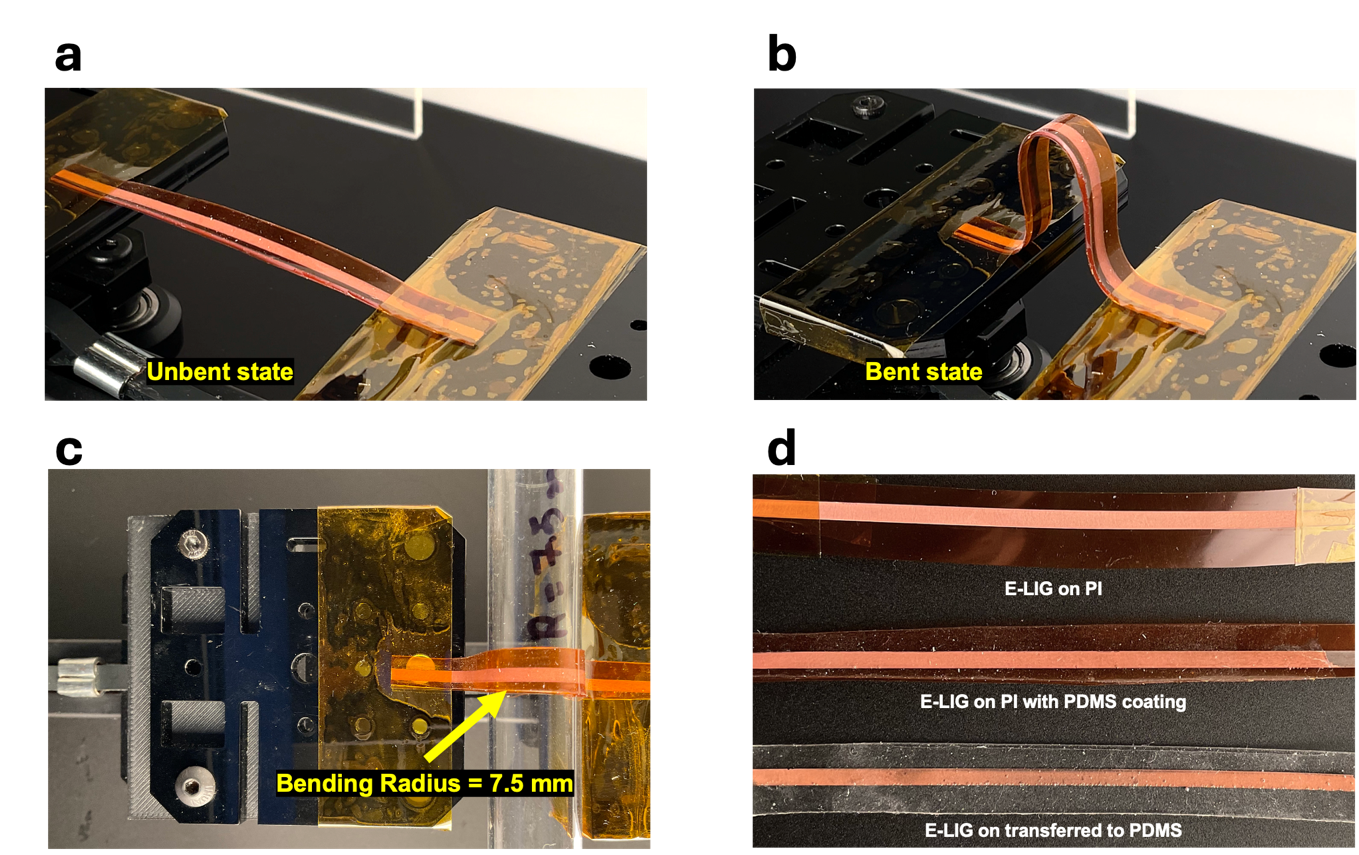


**Figure S14. Bending test setup and results.** (a) Unbent state of the E-LIG sample during the bending test. (b) Bent state of the same sample with a bending radius of 7.5 mm. (c) Top view showing the bending radius (7.5 mm) during testing. (d) Samples after 1000 bending cycles showing no visible damage or delamination for E-LIG on PI, E-LIG on PI with PDMS coating, and transferred E-LIG on PDMS.

**2. 180-Degree Bend Test**

To further evaluate adhesion under extreme deformation, a 180-degree manual bend test was performed on the samples. For Sample 1 (E- LIG on PI) seen in Figure S15a, the copper-plated LIG delaminated at the LIG-PI interface at the edge of the 180-degree bend. This delamination highlights that the LIG-PI bond is weaker compared to the copper-to-LIG bond. The copper remained bonded to the LIG layer, suggesting that the electrochemical bond between copper and LIG is stronger than the physical adhesion between LIG and PI. For Sample 2 (E- LIG on PI with PDMS coating) shown in Figure S15b and Sample 3 (Transferred E- LIG on PDMS) shown in Figure 15c, no delamination or cracking was observed after the 180-degree bend test. The PDMS coating acts as a protective layer, improving the adhesion and preventing delamination during extreme deformation. (Video S12).


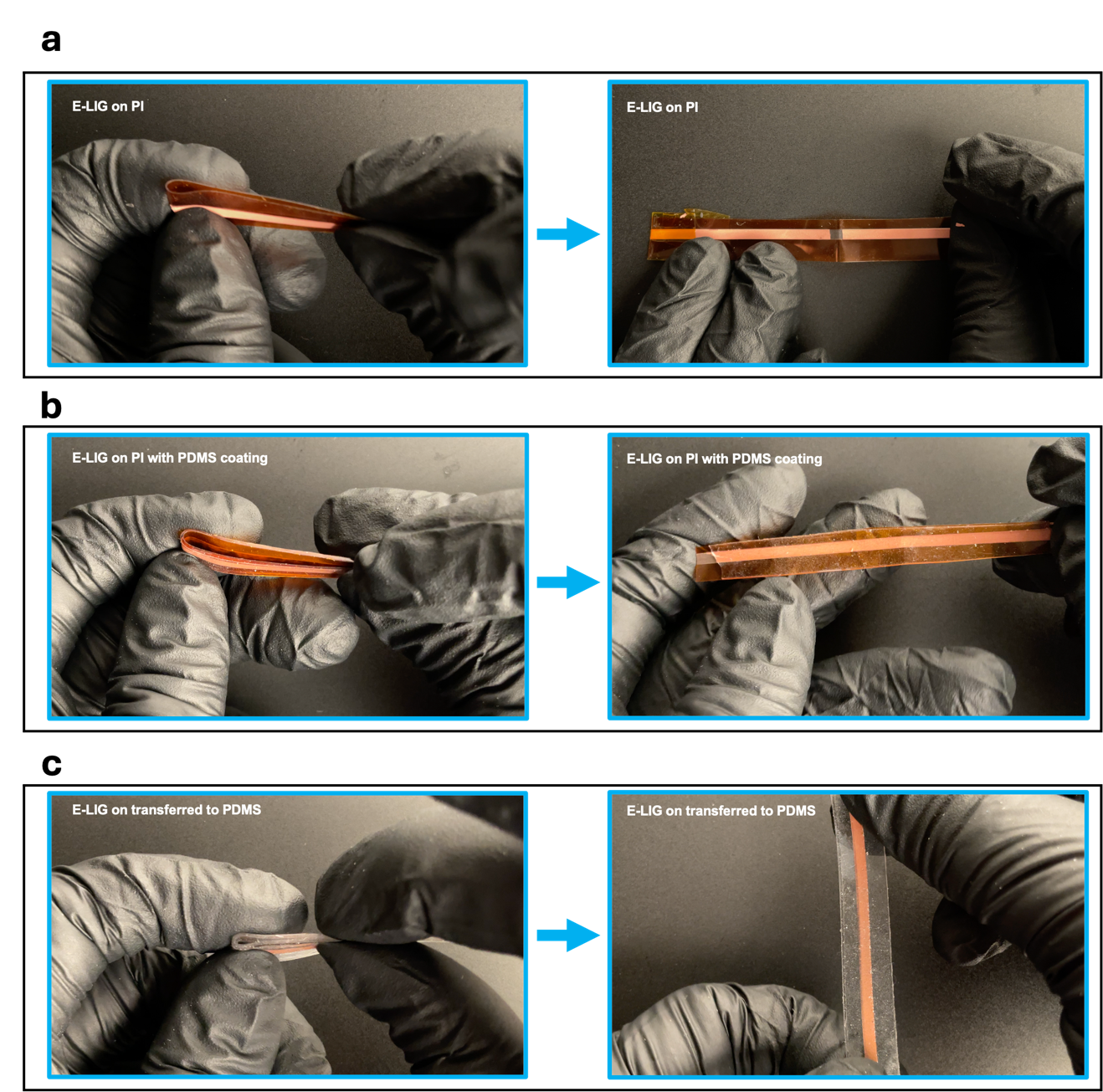


**Figure S15. 180-degree bend test for E-LIG samples.** (a) E-LIG on PI delaminates at the LIG-PI interface after 180-degree test. (b) E-LIG on PI with PDMS coating maintains structural integrity without delamination after the 180-degree bend. (c) Transferred E-LIG on PDMS also shows no delamination or cracking after 180-degree bending.

**3. Strain Test (Up to 60%):**

A strain test was conducted on Sample 3 (Transferred E- LIG on PDMS) to evaluate its performance under tensile deformation. As shown in Figure S16a, the sample was in the unstrained state before testing. The sample was then stretched from 10% to 60% strain using a custom-built strain-testing setup. The 60% strain test is shown in Figure S16b. The sample withstood strains up to 55% without delamination or cracking of the copper plated LIG layer. The failure mode observed at 60% strain, shown in Figures S16c and S16d was a complete mechanical break of the sample (brittle fracture of the substrate), rather than any failure at the copper-LIG interface. This indicates that the copper-to-LIG bond remains intact even under significant tensile deformation.


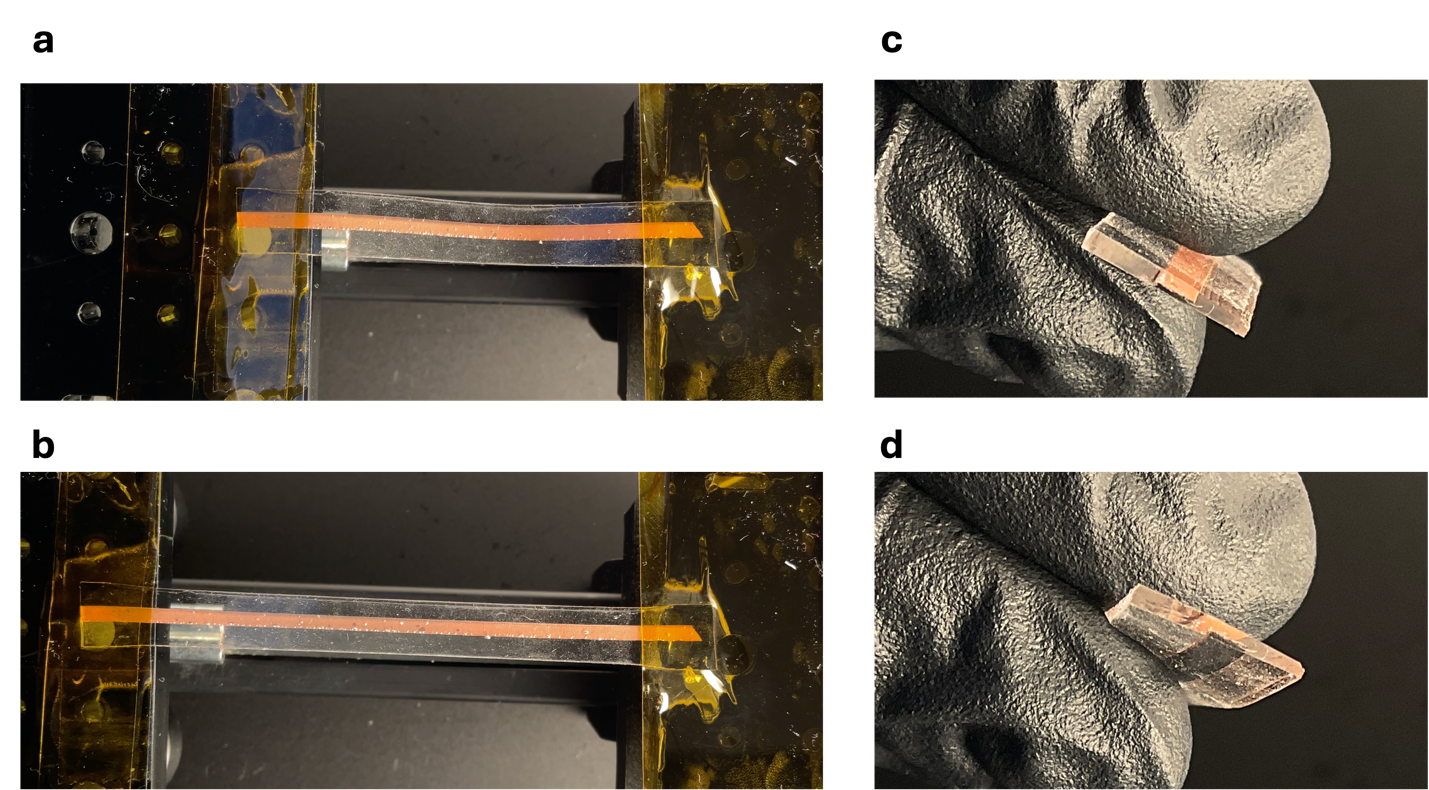


**Figure S16. Strain test**. (a) Unstrained state of the transferred E-LIG on PDMS sample. (b) Strained state at 60% strain, showing no delamination or cracking of the copper-plated LIG layer. (c, d) Failure mode observed after the 60% strain test, showing a complete mechanical break in the PDMS substrate while the copper-to-LIG bond remains intact.

**4. Tape Test**

The adhesion of the copper-plated LIG circuits was further assessed using the tape test. The tape test was conducted to evaluate the adhesion strength of the copper-plated LIG circuits. High-adhesion Kapton tape was applied to the copper-plated surface and peeled off at a controlled angle to assess delamination (Figure S17a). For E-LIG on PI, delamination was observed at the LIG-PI interface, with visible peeling of the copper-plated LIG layer (Figure S17b). In contrast, for Sample of transferred E-LIG on PDMS, no delamination or material removal occurred during the tape test (Figure S17c). These results demonstrate that the PDMS coating effectively enhances adhesion, preventing peeling and improving mechanical robustness against delamination.


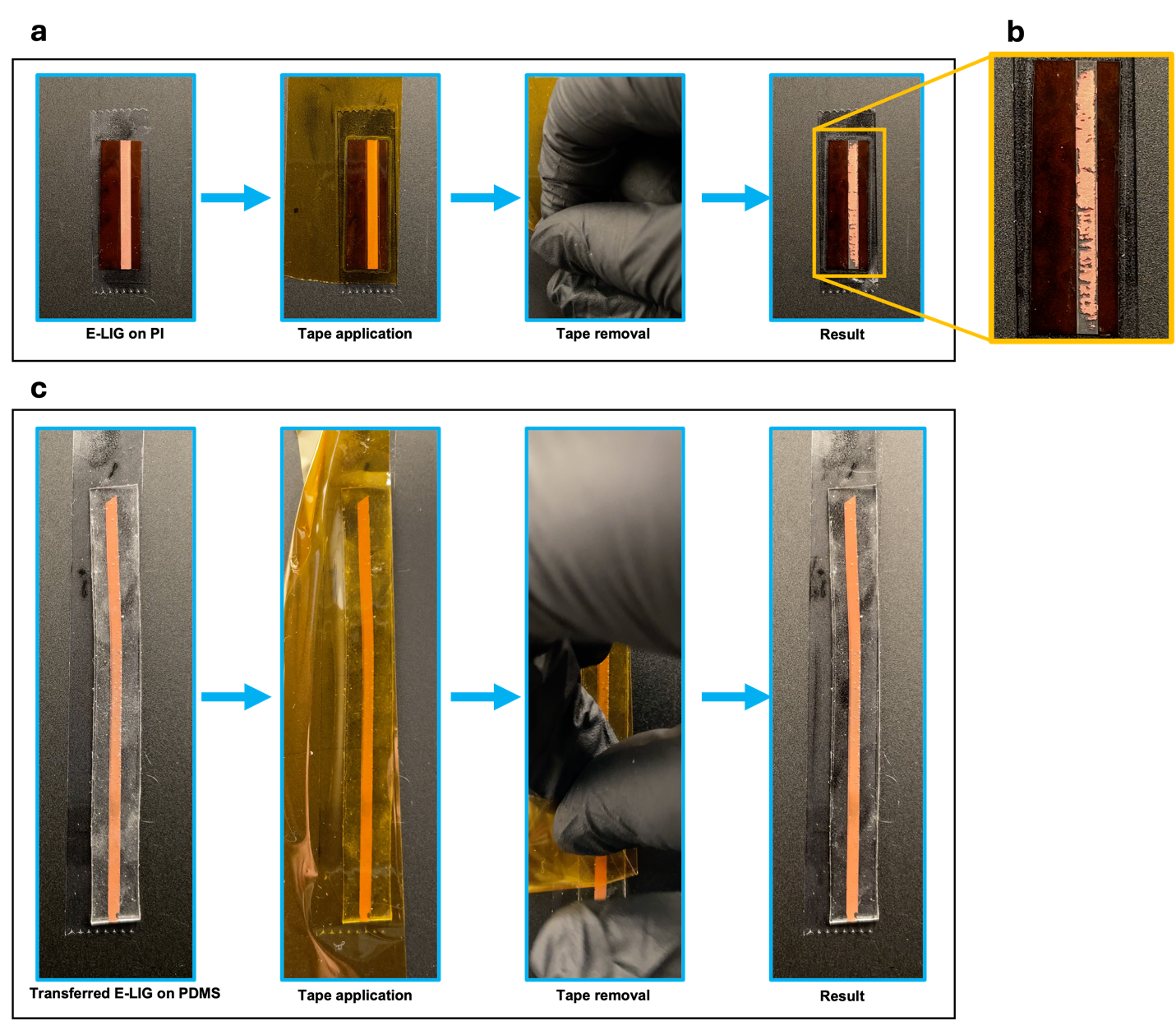


**Figure S17. Tape test.** (a) Sequence images showing the application and peeling of high-adhesion Kapton tape on E-LIG on PI substrate, demonstrating delamination at the LIG-PI interface. (b) Magnified view highlighting the delaminated copper-LIG layer on PI. (c) Sequence images for transferred E-LIG on PDMS, showing no delamination or material removal after the tape test, indicating strong adhesion and mechanical robustness.

The adhesion tests demonstrate that the copper-to-LIG bond is strong and durable across all sample types. However, the LIG-to-PI bond shows weaker adhesion, leading to delamination in some cases, particularly during extreme deformation. This issue can be mitigated by applying a thin PDMS coating on the LIG layer, which improves adhesion and provides mechanical protection against delamination. The results confirm that PDMS, and silicone encapsulation in general is an effective strategy to enhance the robustness and durability of copper-plated LIG circuits for flexible electronic applications. This is particularly important for large-area electronics that are subject to frequent bending, stretching, or mechanical stresses.

**Note S7: Effect of plating voltage on plating uniformity**

The relationship between lower plating voltages and uniform copper coating reported in Figure 3d is further supported here. Comparative experiment with two identical samples of laser-induced graphene (LIG) traces on polyimide was conducted, each measuring 2 mm in width and 8 cm in length. Both samples were plated for 15 minutes but at different voltages: Sample 1 was plated at 0.5 V, while Sample 2 was plated at 1.5 V.

After plating for 15 minutes, we measured the resistance across four equal 2 cm segments of each trace. The resulting segment resistances for Sample 1 were as follows: 0.4 Ω, 0.4 Ω, 0.4 Ω, and 0.5 Ω, yielding an average resistance of 0.425 Ω with a standard deviation of 0.05 Ω, indicating a highly uniform copper coating. In contrast, Sample 2 showed significantly higher variation in resistance across its segments: 3.4 Ω, 2.6 Ω, 0.8 Ω, and 0.4 Ω, with an average resistance of 1.8 Ω and a standard deviation of 1.24 Ω. These results demonstrate that lower plating voltages and current densities yield more uniform copper deposition, likely due to more controlled and gradual ion transfer during the electroplating process, which prevents localized overgrowth while higher current densities lead and larger crystal agglomeration observed with higher voltages which ensures a consistent layer across the electrode ^[20]^.

The optical images (Figure S18 conducted a and Figure S18b) further validate these observations, showing a visually smoother and more consistent copper coating for the sample plated at 0.5 V compared to the rougher and more uneven coating observed at 1.5 V. This experimental evidence directly supports our statement in Figure 3d regarding the advantages of using lower plating voltages for achieving uniform coatings in flexible electronic applications.

Table S5:

| **Sample #** | **Plating Time (min)** | **Plating Voltage (V)** | **R _Segment 1_ (Ω)** | **R _Segment 2_ (Ω)** | **R _Segment 3_ (Ω)** | **R _Segment 4_ (Ω)** | **Mean ± SD** |
| --- | --- | --- | --- | --- | --- | --- | --- |
| 1 | 15 | 0.5 | 0.4 | 0.4 | 0.4 | 0.5 | 0.425 ± 0.05 |
| 2 | 15 | 1.5 | 3.4 | 2.6 | .8 | .4 | 1.8 ±1.24 |


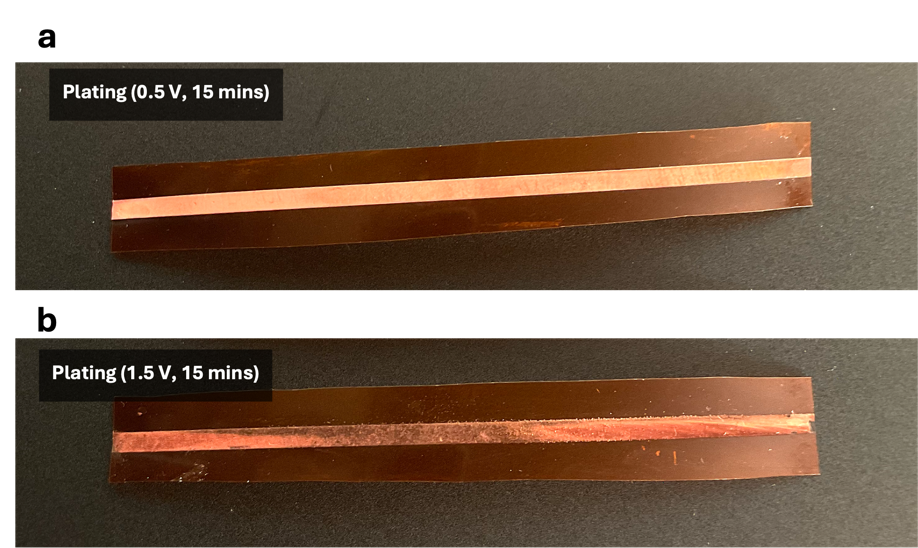


**Figure S18.** Optical images of two E-LIG electrodes plated under different voltages. (a) Sample 1 plated at 0.5 V for 15 minutes shows a uniform copper coating, as evidenced by the consistent color across the electrode. (b) Sample 2 plated at 1.5 V for 15 minutes exhibits visible roughness, overgrowth and uneven copper deposition conducted.

**Note S8: Effect of via surface smoothness on electrode resistance**

It was observed that the generated LIG via had a surface that is rough in its nature. To study the potential impact of via surface roughness on resistance, and the subsequent electroplating quality, additional experiments were conducted. We found that the primary cause of roughness in the via is the ablation process during laser-induced graphene (LIG) formation. When the laser ablates the polyimide to create the via, rough, flaky edges and loose graphene debris are generated at the rim of the hole. These loose flakes and debris subsequently get plated during the electroplating process, resulting in a rough copper surface inside the via.

To address this issue, we incorporated a cleaning step before plating to remove the debris and smoothen the via surface. After creating the vias via laser ablation, we rinsed the samples with isopropyl alcohol (IPA) to clean the ablated holes, removing loose flakes and debris. Upon electroplating, the cleaned vias exhibited a visibly smoother copper surface compared to non-cleaned vias, as shown in **Figure S19.** The comparison demonstrates the effectiveness of this cleaning method in improving via surface quality.

In terms of electrical conductivity, we observed that the roughness did not significantly affect the resistance of the via in our experiments. Both cleaned and non-cleaned vias yielded similar resistance values, indicating that the primary requirement for conductivity is ensuring sufficient copper coverage to establish electrical contact across the via. However, we believe that achieving smoother vias is still advantageous for preventing clogged vias, since rough or clogged vias can inhibit continuous plating to the opposite side of the substrate, potentially causing incomplete or inconsistent electroplating. In summary, while the surface roughness of the via did not significantly impact the resistance in our tests, we believe that implementing a cleaning step before plating enhances the overall quality of the via by preventing clogging and ensuring a more continuous plating process.


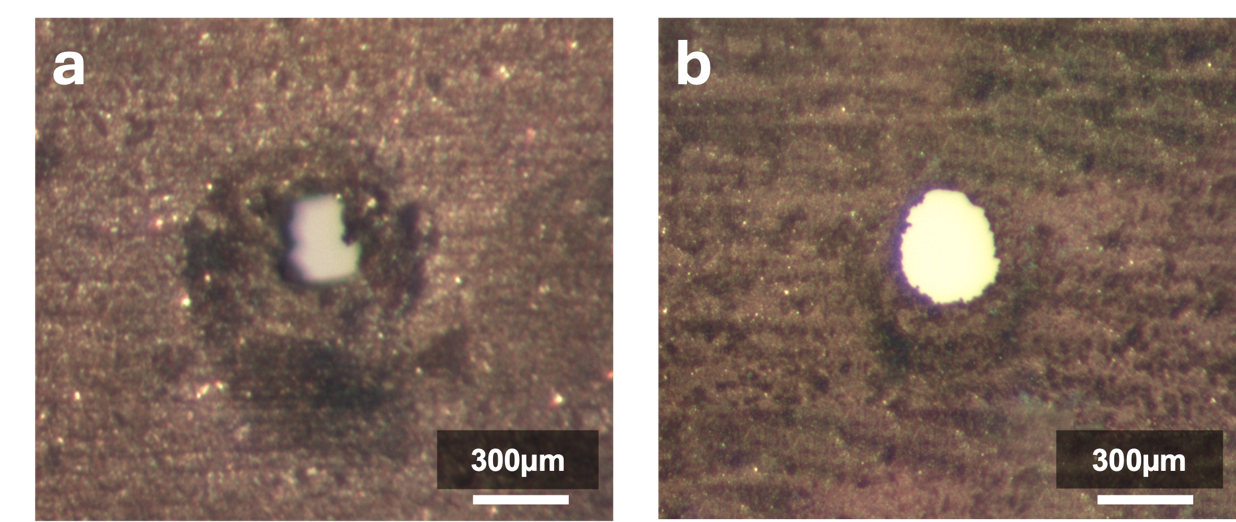


**Figure S19.** Microscopic images of copper-plated vias on polyimide. (a) Non-cleaned via with rough copper deposition due to debris from laser ablation. (b) Cleaned via after rinsing with IPA, showing a smoother surface.

**Supporting Videos Descriptions:**

**Video S1: General E-LIG Process Flow**

- Title: Step-by-Step Overview of E-LIG Fabrication
- Description: This video presents an overview of the E-LIG fabrication process, demonstrating each step in the process.

**Video S2: Double-Sided Fabrication Process Flow**

- Title: Double-Sided E-LIG Circuit Fabrication
- Description: This video shows the double-sided E-LIG fabrication process, including the formation of laser-patterned vias to connect both sides.

**Video S3: Strain Sensor LED Intensity Control**

- Title: *Controlling LED Intensity with* Strain *Sensor*
- Description: This video demonstrates the control of a LED brightness based on real-time strain sensor readings. Arduino IDE was used for programming, showing the sensor’s closed-loop response.

**Video S4: Real-Time Data Plotting for Strain Sensor**

- Title: *Real-Time Data Plotting of Strain Sensor Performance*
- Description: Real-time data plotting of the strain sensor is demonstrated using the Arduino IDE and Processing for basic plotting, capturing live resistance changes correlated with the strain.

**Video S5: Graphical Data Visualization of Strain Sensor Performance**

- Title: *Graphical Data Visualization of Strain Sensor*
- Description: Advanced data visualization of strain sensor is shown using Processing software. The dynamic graphical interface includes both an extending spring and a defoming grid representation based on strain-induced resistance changes.

**Video S6: Pressure Sensor Demonstration & LED control**

- Title: *Programming the Arduino board and uploading a blinking LED code*
- Description: This video demonstrates the initial Arduino programming setup using Arduino IDE, where a blinking LED code is uploaded to the PCB to validate the board’s basic functionality before integrating the pressure sensor.
- The video also includes LED brightness control.. Arduino IDE was used for programming, and the brightness of the LED changes proportionally to the pressure applied.

**Video S7: Real-Time Data Plotting for Pressure Sensor**

- Title: *Real-Time Data Plotting of Pressure Sensor Performance*
- Description: Real-time data logging of the pressure sensor is shown using basic plotting via Arduino IDE and Processing. The pressure readings are captured and displayed continuously during the test as a line graph.

**Video S8: Graphical Data Visualization of Pressure Sensor Performance**

- Title: *Graphical Data Visualization of Pressure Sensor*
- Description: This video demonstrates advanced graphical data visualization using the Processing software. It includes a dynamic bar graph and an expanding circle visual representation, correlating with pressure intensity.

**Video S9: Programmed Activation of Heater using E-LIG Circuit**

- Title: *Heater Activation with Phototransistor Mapping*
- Description: This video demonstrates the programmed activation of the thin-film heater. The heater is activated in a closed-loop manner based on a phototransistor threshold programmed in the Arduino IDE. The thermal camera captures temperature distribution, and the heater responds once the threshold is triggered, showing integrated closed-loop functionality.

**Video S10: Programmed Activation of Electrothermal Actuator using E-LIG Circuit**

- Title: *Electrothermal Actuator Activation and Operation*
- Description: This video shows the programmed one-time actuation of the electrothermal actuator, captured using a thermal camera. The actuator’s bending motion is triggered by voltage inputs, demonstrating its capability for precise shape transformation based on programmed activation.

**Video S11: On-Demand Circuit Repairability**

- Title: Demonstration of On-Demand Circuit Repair
- Description: This video demonstrates the repairability feature of the E-LIG process. It shows how broken connections can be restored by re-lasing the substrate, allowing graphene to re-form and repair the circuit on demand.

**Video S12: Bending and Strain Tests of E-LIG Electrodes**

- **Title**: Bending and Strain Tests of E-LIG Electrodes
- **Description**: This video demonstrates the mechanical robustness of copper-plated E-LIG. Bending Test: No delamination or cracks after 1000 cycles at a 7.5 mm radius.Strain Test: No interface failure up to 55% strain, with failure occurring as a mechanical break at 60%.

**References**

[1] “Screen Printing vs. Direct Ink Writing for Printed Electronics,” can be found under https://www.voltera.io/blogscreen-printing-direct-ink-writing-printed-electronics, **n.d.**

[2] S. B. Balani, S. H. Ghaffar, M. Chougan, E. Pei, E. Şahin, *Results in Engineering* **2021**, *11*, 100257.

[3] A. A. Alsharif, J. M. Aviles, F. M. Zechel, N. A. Alsharif, N. El-Atab, *VIEW* **2024**, *5*, 20240008.

[4] D. Munoz-Martin, C. F. Brasz, Y. Chen, M. Morales, C. B. Arnold, C. Molpeceres, *Applied Surface Science* **2016**, *366*, 389.

[5] “Design Rule Check - PCB Prototype the Easy Way - PCBWay,” can be found under https://www.pcbway.com/pcb_prototype/PCB_Design_Rule_Check.html, **n.d.**

[6] S. F. Kamarudin, N. H. Abdul Aziz, H. W. Lee, M. Jaafar, S. Sulaiman, *Advanced Materials Technologies* **2024**, *9*, 2301875.

[7] Y.-G. Park, I. Yun, W. G. Chung, W. Park, D. H. Lee, J.-U. Park, *Advanced Science* **2022**, *9*, 2104623.

[8] S. Khan, L. Lorenzelli, R. S. Dahiya, *IEEE Sensors Journal* **2015**, *15*, 3164.

[9] “Fabrication and Characterization of Flexible Spray-Coated Antennas | IEEE Journals & Magazine | IEEE Xplore,” can be found under https://ieeexplore.ieee.org/document/8493481, **n.d.**

[10] J. Lin, Z. Peng, Y. Liu, F. Ruiz-Zepeda, R. Ye, E. L. G. Samuel, M. J. Yacaman, B. I. Yakobson, J. M. Tour, *Nat Commun* **2014**, *5*, 5714.

[11] A. Raouafi, I. Diedhiou, A. H. Almarri, N. Raouafi, *emergent mater.* **2024**, *7*, 2945.

[12] A. Velasco, Y. K. Ryu, A. Hamada, A. De Andrés, F. Calle, J. Martinez, *Nanomaterials* **2023**, *13*, 788.

[13] Q. Chen, D. Wu, Z. Chen, H. Yu, Q. Chen, D. Sun, in *2021 IEEE 16th International Conference on Nano/Micro Engineered and Molecular Systems (NEMS)*, IEEE, Xiamen, China, **2021**, pp. 1650–1654.

[14] J. de la Roche, I. López-Cifuentes, A. Jaramillo-Botero, *Carbon Lett.* **2023**, *33*, 587.

[15] A. Behrent, C. Griesche, P. Sippel, A. J. Baeumner, *Microchim Acta* **2021**, *188*, 159.

[16] Q. Fu, W. Li, F. E. Kruis, *Nanotechnology* **2023**, *34*, 225601.

[17] L. Cheng, C. S. Yeung, L. Huang, G. Ye, J. Yan, W. Li, C. Yiu, F.-R. Chen, H. Shen, B. Z. Tang, Y. Ren, X. Yu, R. Ye, *Nat Commun* **2024**, *15*, 2925.

[18] K. Ratautas, A. Jagminienė, I. Stankevičienė, M. Sadauskas, E. Norkus, G. Račiukaitis, *Results in Physics* **2020**, *16*, 102943.

[19] C. Zhao, J. Wang, *physica status solidi (a)* **2014**, *211*, 2878.

[20] M. Kamel, A. A. El_moemen, S. Rashwan, A. Bolbol, *Metall* **2017**, *71*, 7.
